# The effects of protein interactions, gene essentiality and regulatory regions on expression variation

**DOI:** 10.1186/1752-0509-2-54

**Published:** 2008-06-26

**Authors:** Linqi Zhou, Xiaotu Ma, Fengzhu Sun

**Affiliations:** 1 Molecular and Computational Biology Program, Department of Biological Sciences, University of Southern California, Los Angeles, CA 90089-2913, USA

## Abstract

**Background:**

Identifying factors affecting gene expression variation is a challenging problem in genetics. Previous studies have shown that the presence of TATA box, the number of *cis*-regulatory elements, gene essentiality, and protein interactions significantly affect gene expression variation. Nonetheless, the need to obtain a more complete understanding of such factors and how their interactions influence gene expression variation remains a challenge. The growth rates of yeast cells under several DNA-damaging conditions have been studied and a gene's toxicity degree is defined as the number of such conditions that the growth rate of the yeast deletion strain is significantly affected. Since toxicity degree reflects a gene's importance to cell survival under DNA-damaging conditions, we expect that it is negatively associated with gene expression variation. Mutations in both *cis*-regulatory elements and transcription factors (TF) regulating a gene affect the gene's expression and thus we study the relationship between gene expression variation and the number of TFs regulating a gene. Most importantly we study how these factors interact with each other influencing gene expression variation.

**Results:**

Using yeast as a model system, we evaluated the effects of four separate factors and their interactions on gene expression variation: protein interaction degree, toxicity degree, number of TFs, and the presence of TATA box. Results showed that 1) gene expression variation is negatively correlated with the protein interaction degree in the protein interaction network, 2) essential genes tend to have less expression variation than non-essential genes and gene expression variation decreases with toxicity degree, and 3) the number of TFs regulating a gene is the most important factor influencing gene expression variation (R^2 ^= 8–14%). In addition, the number of TFs regulating a gene was found to be an important factor influencing gene expression variation for both TATA-containing and non-TATA-containing genes, but with different association strength. Moreover, gene expression variation was significantly negatively correlated with toxicity degree only for TATA-containing genes.

**Conclusion:**

The finding that distinct mechanisms may influence gene expression variation in TATA-containing and non-TATA-containing genes, provides new insights into the mechanisms that underlie the evolution of gene expression.

## Background

Gene expression variation has been studied on three different levels: single cells across a common environment [1], within one species across a variety of different environments [2,3], and across different species/strains, which is often referred to as evolutionary variation [4-8]. In this paper, we study genetic factors affecting gene expression variation within one species across many different environmental conditions. Broadly, the genetic factors affecting gene expression primarily include the binding of regulatory proteins to *cis*-elements in the upstream of the gene, as well as physical and genetic interactions with other genes. With the availability of many gene expression profiles, protein interaction networks, and gene regulatory networks, it is now possible to study how gene expression variation is associated with both network features and genomic factors. In the case of protein interaction networks, interaction degree, i.e., the number of interacting partners of a given protein, is one of many factors. The presence or absence of TATA box and the number of transcription factors (TF) regulating a gene provide examples of genomic factors influencing gene expression variation.

Many studies have focused on individual factors affecting gene expression variation. For instance, Newman et al. [1] developed an experimental technique to study protein expression noise in single cells and showed that chromosomal distance to other genes and mRNA-half life are associated with expression noise. However, they did not find a relationship between protein expression noise and protein-protein interactions. Recently, using a more complete interaction dataset, Batada et al. [9] found that protein expression variation is negatively correlated with interaction degree when protein abundance was controlled using the data in Newman et al. [1]. This relationship continues to hold within the viable genes. Several groups investigated mRNA expression variation within species. For example, Nelson et al. [2] studied the relationship between the number of tissues or body parts (expression variation), where the gene is expressed, and gene spacing in *C. elegans *and *D. melanogaster*. They found that gene expression variation increases in relation to the intergenic distances between genes. Walther et al. [3] found a positive correlation between the frequency of a gene's differential expression and the number of *cis*-regulatory elements of that gene in *A. thaliana*. Furthermore, several groups have studied gene expression variation, also known as evolutionary variation, across different species/strains. Using gene expression data from several yeast species [7], as well as from different strains derived from mutation-accumulation experiments [6], it was found, for instance, that the interspecies/interstrain variation of gene expression is significantly correlated with the presence/absence of the TATA box in the promoter region. Lemos et al. [4,5] studied the effect of protein-protein interactions and protein length on evolutionary variation (variation among strains in a species). They found that evolutionary variation is negatively correlated with protein-protein interactions in *Saccharomyces cerevisiae *or *Drosophila melanogaster *[4] and negatively correlated with protein length in *Drosophila melanogaster *[5]. These studies highlighted the importance of protein interactions and gene regulatory regions on gene expression variation.

Only a few studies, however, have integrated such different data sources in a way that collectively identifies and interprets the key factors affecting gene expression variation. Therefore, we conducted studies of proteomic and genomic factors marginally and collectively influencing gene expression variation across different perturbation conditions within one species: yeast.

Protein interactions play an important role in gene expression variation. Protein-protein interactions are key biological events in a living cell, and proteins in a cell interact with each other to perform certain functions. High throughput technologies, including yeast two-hybrid systems and mass spectrometry, have generated a large amount of protein interactions in yeast. Computational methods have also been developed to study the reliability of the observed interactions [10,11] and to build reliable protein interaction networks. These efforts have resulted in the development of several protein interaction databases, albeit with differing degrees of reliability, including MIPS [12], DIP [13] and BioGrid [14]. From an evolutionary point of view, the expression profiles of neighboring genes of a target gene in a protein interaction network may put some constraints on the target gene's expression. Thus, in a protein interaction network, the interacting partners of a specific protein can affect the corresponding gene's expression. Therefore, protein physical interaction degree, i.e., the number of interacting partners of a given protein, can significantly affect gene expression variation. In the present study, we show that gene expression variation decreases with protein interaction degree and that protein interaction degree accounts for 1–2% of the expression variation in model organism yeast, a result consistent with previous studies [4,5].

Another key factor affecting gene expression variation is gene essentiality. Genes can be classified into essential and non-essential genes based on the fitness phenotype of the yeast cell when the gene is deleted under normal growth conditions [15]. Essential genes are those that, when deleted, will render the yeast cell non-viable. Non-essential genes can be further classified into no-phenotype and toxicity-modulating genes based on the fitness phenotype of yeast cell when the gene is deleted under the conditions of four DNA-damaging treatments [16]. Specifically, we define a gene's toxicity modulation degree as the number of DNA-damaging treatments significantly affecting the deletion strain's fitness (toxicity modulation degree = 0 (no phenotype), 1, 2, 3, and 4). The higher the toxicity modulation degree, the more important the gene is in relation to cell survival. Therefore, toxicity degree gives a quantitative measurement of a gene's importance to yeast cell survival. We measure a gene's functional importance in relation to cell survival by the essentiality of the essential genes and the toxicity modulation degree of non-essential genes. Since the expression of genes important for cell survival are generally stable under many different stimuli and cannot fluctuate extensively, we hypothesize and show that expression variation of essential genes is lower than that of non-essential genes and decreases with toxicity degree within non-essential genes.

The number of *cis*-elements has been shown to be positively associated with gene expression variation [6]. The number of *cis*-elements is usually approximated using computational approaches and many contain false positive and negative predictions. Theoretically, a given gene's expression pattern can become increasingly complex with the increasing number of transcription factors that regulate this gene, either directly or indirectly. In this study, we hypothesize that the number of TFs is a significant predictor of expression variation and show that the number of TFs regulating a gene (hereinafter referred to as 'number of TFs') accounts for 8–14% of its expression variation, much higher than that can be explained by the number of *cis*-elements (0.3–1.7%). This implies the importance of indirect trans-effect on expression variation.

The TATA box is a conserved element in the eukaryotic promoter region and is usually bound by TATA-binding proteins. The presence of TATA box has been shown to be one of the most important factors contributing to gene expression variation [6,7]. Further analysis of the individual genomic and proteomic factors affecting gene expression indicates that there might be two distinct mechanisms that specifically influence gene expression variation of TATA-containing and non-TATA-containing genes. Most importantly, we show that significant negative correlation between expression variation and toxicity degree is only present for TATA-containing genes and that toxicity degree accounts for 1.3–2.6% of the expression variation. In contrast, the relationship between expression variation and toxicity degree is absent for non-TATA-containing genes. The fact that TATA-containing genes are enriched in stress-related genes [17] may explain this difference. Although the number of TFs is significantly positively correlated with expression variation for both TATA- and non-TATA-containing genes, the association strength is higher for non-TATA containing genes than for TATA-containing genes. These results imply that the mechanism influencing TATA-containing gene expression variation is much more complicated than that in non-TATA-containing genes. For example, TATA-containing genes were found more likely to be epigenetic regulated [17,18]. Thus, this study gives a more complete analysis of factors and their interaction affecting gene expression variation than may be found in previous studies.

## Results and Discussion

We present our results based on the MIPS protein physical interaction data [12] and the yeast gene expression profiles under 40 Ca and Na exposure conditions [19]. The results based on three interaction datasets (MIPS [12], DIP [13], and BioGrid [14]) and four other gene expression datasets (chemostat (nutritional stress) [20], environmental stress [21], oxidative stress [22], and a combined gene expression dataset over more than 1,500 conditions [7]) are given in the Additional Files 1 and 2. We study the expression data individually in order to minimize the variation among different laboratories. By doing so, we can also confirm whether the results based on different gene expression data are consistent. Consistency of results using a variety of different datasets adds confidence to the conclusions. In this manuscript, we use genes and proteins interchangeably. We declare statistical significance if a p-value is less than 0.05 without adjusting for multiple comparisons. In this study, we conducted an exploratory study of factors affecting gene expression variation. As in many epidemiological studies, we did not adjust p-values for multiple comparisons. Therefore, some of our findings need to be further tested in other datasets.

### Gene expression variation versus protein interaction degree

**Figure 1:**
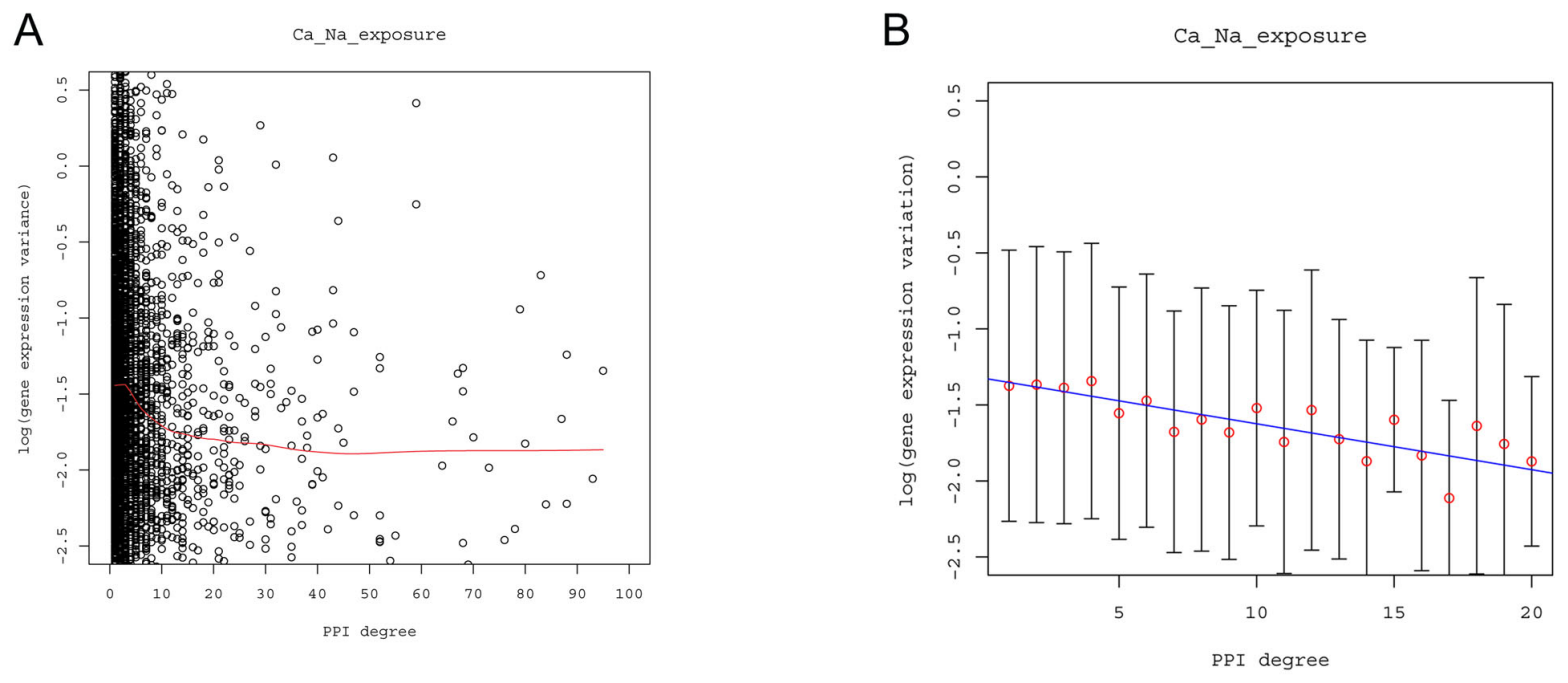
**Gene expression variation is negatively correlated with protein interaction degree**. The x-axis represents protein physical interaction degree, and the y-axis represents gene expression variation. A) The LOWESS fit to the gene expression variation. B) Bar-plot of the expression variation of all the genes with a given protein interaction degree together with the linear regression fit to the gene expression variation in relation to the interaction degree. The linear coefficient β = -0.0302, R^2 ^= 1.41%, and p-value = 9.704e-14. The red dots are the mean expression variation of the genes given the protein physical interaction (PPI) degree. The bar represents the standard deviation of the gene expression variation given PPI degree. To keep the same scale for gene expression variation across the figures, the range of the y-axis is -2.5 to 0.5.

We measured gene expression variation by the logarithm of the variance of the expression levels of each gene across the 40 Ca and Na exposure conditions [19]. We then studied the relationship between the expression variation and protein interaction degree using the LOWESS function in R [23] to fit the data. On these bases, it was obvious that gene expression variation decreases with the degree of protein physical interaction (Figure 1A). The decreasing trend is especially significant when the interaction degree is relatively low (≤ 20). In contrast, when the interaction degree is greater than 40, the decreasing trend is not as obvious. Biologically, this may be explained by the fact that gene expression variation stabilizes when the interaction degree is above a given threshold. Another potential explanation is that interactions between proteins of high degrees are simply less reliable [24]. The large number of less reliable interactions for proteins with high degrees can skew the true relationship between gene expression variation and interaction degrees. Since the true underlying mechanism of this phenomenon is not clear, we limited our further analysis to proteins with an interaction degree of no more than 20. Protein physical interaction degree has been found to be negatively associated with gene expression variation for single cells [9] and evolutionary expression variation [4,5] between different strains/species. Our result on the relationship between gene expression variation and interacting degree within *S. cerevisiae *is consistent with their findings.

Accordingly, we then used linear regression to fit the expression variation for proteins with a maximal physical interaction degree of 20:

*v *= *α *+ *βd*

where *v *is the gene expression variation and *d *is the interaction degree. α and β are parameters. The fitted line and the corresponding bar-plot for the expression variation are shown in Figure 1B. The gene expression variation is significantly negatively correlated with the protein interaction degree (≤ 20) (R^2 ^= 1.41%, β = -0.0302, p-value = 9.704e-14). The negative correlation between expression variation and interaction degree implies that protein with high interaction degrees do not tolerate extensive expression variation and such protein need more precise control on gene expression for an organism to function normally.

### Gene expression variation versus essentiality, toxicity modulation, and interaction degrees

**Figure 2:**
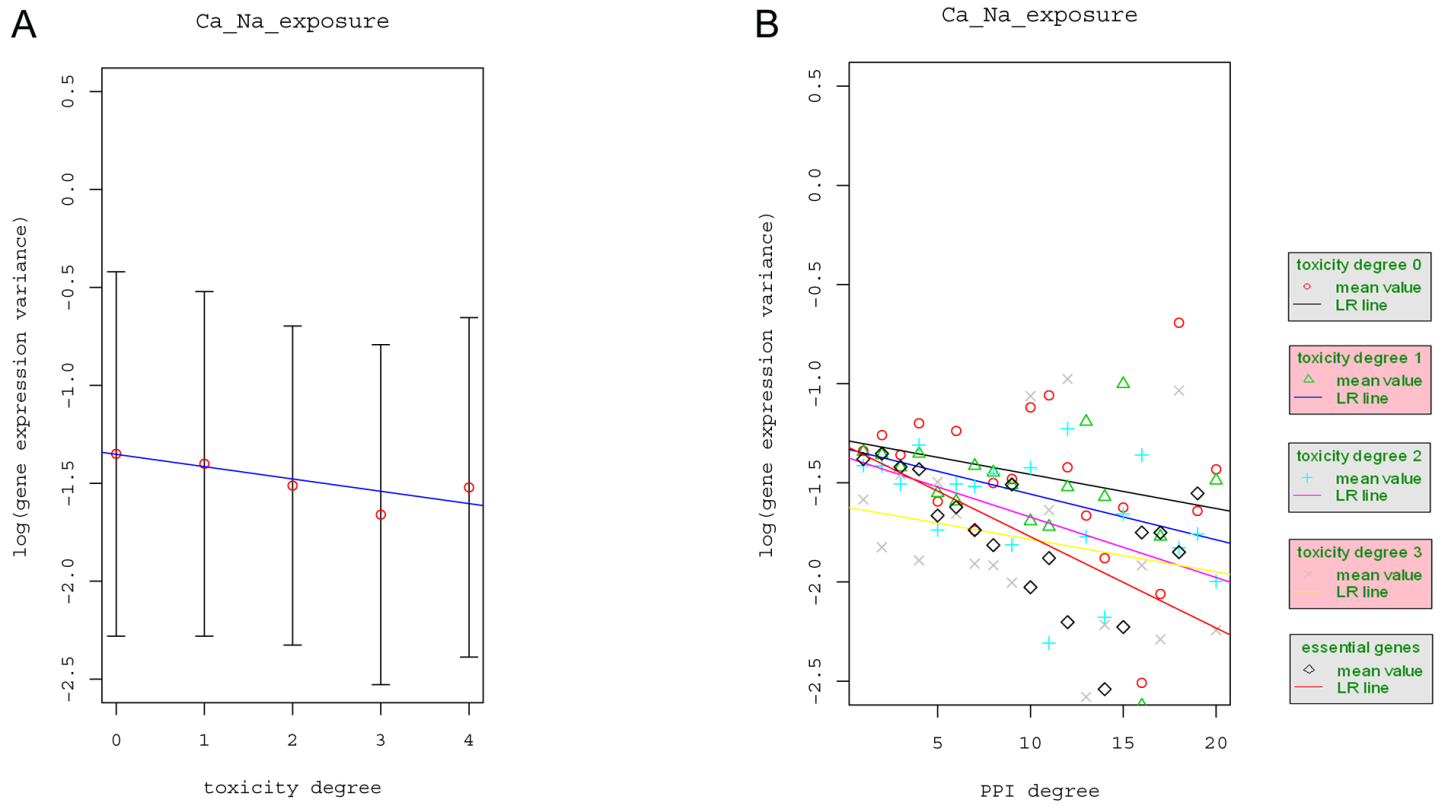
**The effect of essentiality, toxicity degree, and protein interaction degree on gene expression variation**. A) Bar-plot of the expression variation of all the genes with a given toxicity degree together with the linear regression fit to the expression variation of the genes in relation to the toxicity degree. The linear coefficient β = -0.0629, R^2 ^= 0.75%, and the p-value = 4.73e-08. B) The mean expression variation and the linear regression fit to the expression variation with respect to PPI degree for non-essential genes stratified according to toxicity degree and for the essential genes. The β values are -0.0172, -0.0230, -0.0304, -0.0164 and -0.0460 for toxicity degree 0, 1, 2, and 3, and the essential genes, respectively. The corresponding p-values are 0.0313, 0.0141, 0.0011, 0.2248 and 0.0013, respectively. R^2 ^is 0.31%, 0.75%, 2.75%, 0.8% and 4.41%, respectively. The labels are the same as those in Figure 1.

As noted above, we divided genes into two classes: essential and non-essential genes. Essential genes are less likely to be perturbed than non-essential genes, as significant perturbations of essential genes will, for example, render the yeast cell non-viable. We further classified the non-essential genes into five groups (no phenotype, 0, and toxicity-modulating proteins with degrees 1, 2, 3, and 4, respectively) according to the cell's fitness phenotype changes under four DNA-damaging agents (methylating agent methyl methanesulfonate (MMS), the bulky alkylating agent 4-nitroquinoline-N-oxide (4NQO), the oxidizing agent tert-butyl hydroperoxide (t-BuOOH), and 254-nm UV radiation) when the non-essential genes are knocked out [16]. Since toxicity degree reflects the functional importance of genes in relation to cell survival under several DNA-damaging perturbations, we expected that gene expression variation would decrease as the toxicity degree increases. Since the number of genes with toxicity degree 4 was small (n = 32), we combined them with the group having toxicity degree 3. We referred to the essential genes as the group with toxicity degree 4. Figure 2A shows the bar-plot and the linear regression fit of the gene expression variation with respect to toxicity degree. Indeed, a significant negative association between gene expression variation and toxicity degree was observed (R^2 ^= 0.75%, β = -0.0629, p-value = 4.73e-08). In the study of the relationship between gene essentiality and evolutionary expression variation conducted by Tirosh and Barkai [8] and Choi et al. [25], they found that essential genes tend to have lower variation than non-essential genes. This is consistent with our result which demonstrates that the variation in essential genes (toxicity degree = 4) is lower than that of non-essential genes (combine the proteins with toxicity degree = 0, 1, 2, 3) (p-value = 0.027). However, our study differs from these two studies in that we further classified the genes according to their toxicity degree and found negative association between gene expression and toxicity degree.

We observed a positive correlation between interaction degree and toxicity degree (data not shown) and, therefore, asked whether the observed negative correlation between gene expression variation and interaction degree is, conversely, caused by the positive correlation between interaction degree and toxicity degree. We consequently studied the relationship between gene expression variation and protein interaction degree within gene groups stratified according to their toxicity degrees (Figure 2B). Using Ca and Na exposure gene expression data [19], we found a significant decreasing trend of gene expression variation with respect to interaction degree in all the strata except for the one with toxicity degree 3. The corresponding (R^2^, β, p-value) are (0.31%, -0.017, 0.03), (0.75%, -0.023, 0.014), (2.75%, -0.030, 0.001), (0.8%, -0.016, 0.2248), and (4.41%, -0.046, 0.001) for toxicity degrees 0, 1, 2, 3 and 4, respectively. The fraction of expression variation explained by the protein interaction degree seems to increase as the toxicity degree increases.

In our analyses, both toxicity and protein interaction degrees are negatively associated with gene expression variation. Hence, the more important a gene is to the survival of the yeast cell, the less variation there is in its expression levels across many different conditions. Similarly, the higher the interaction degree of a gene, the more stability is observed in its expression levels. Biologically, a gene is important to the cell's survival since it participates in many important biological processes. Any perturbation of this gene's expression will likely cause deleterious effect to the corresponding biological process and thus renders the cell non-viable. An evolutionary consequence of this hypothesis is that genes important to cell survival appear to have robust expression levels.

### Expression variation versus gene regulatory regions: TATA box, number of TFs, and toxicity degree

**Figure 3:**
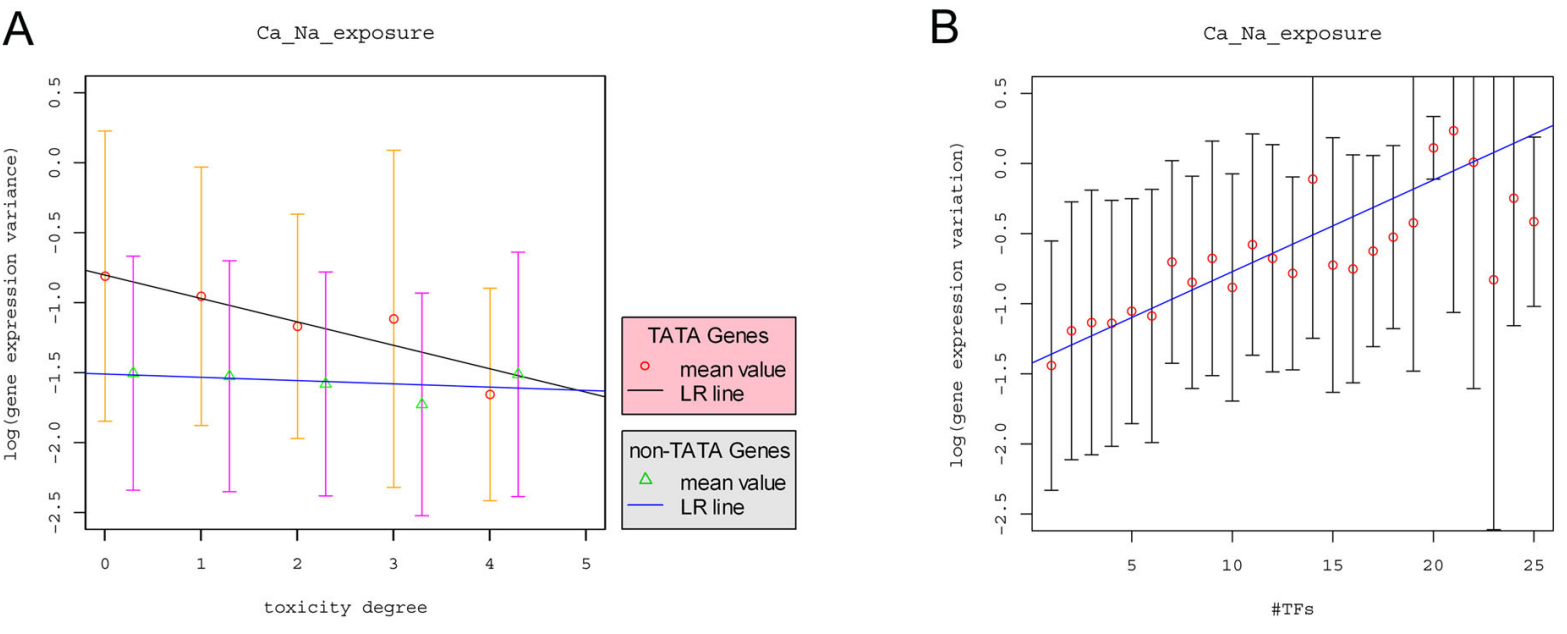
**The effect of TATA box, number of TFs, and toxicity degree on gene expression variation**. A) The relationship between expression variation and toxicity degree stratified by the presence/absence of the TATA box (R^2 ^= 2.59%, β = -0.1674, p-value = 7.174e-06 for the TATA-containing gene set; R^2 ^= 0.13%, β = -0.0234, p-value = 0.0413 for the non-TATA-containing gene set). B) The relationship between expression variation and the number of TFs up to 25 (R^2 ^= 8.28%, β = 0.0654, p-value < 2.2e-16). The labels are the same as those in Figure 1.

Previous studies have established the relationship between gene expression variation and the regulatory regions, including the presence/absence of TATA box [6,7], the length of intergenic regions [2], and the number of *cis*-regulatory elements [3]. We therefore asked if the observed relationship between gene expression variation and toxicity degree are the same for TATA-containing genes and non-TATA-containing genes. To accomplish this goal, we first stratified the yeast genes based on the presence/absence of TATA boxes and reanalyzed the relationship between gene expression variation and toxicity degree. Consistent with previous findings [6,7], it is clear that the gene expression variation of TATA-containing genes is much higher than that of non-TATA-containing genes for each fixed toxicity degree (p-value < 2.2e-16). Significant negative association between gene expression variation and toxicity degree was observed for the TATA-containing group (Figure 3A) (R^2 ^= 2.59%, β = -0.1674, p-value = 7.174e-06). However, the association between expression variation and toxicity degree for the non-TATA-containing group is only marginally significant (R^2 ^= 0.13%, β = -0.02338, p-value = 0.0413). Consistent with the other two gene expression datasets, chemostat [20] and environmental stress [21], as well as the combined gene expression data from [7] (see Additional File 2: Supplementary Table 7), such a highly significant negative relationship between gene expression variation and toxicity degree could be observed in TATA-containing genes, but not for the non-TATA-containing genes. Thus, the effect of toxicity degree on gene expression variation is different for TATA-containing genes versus non-TATA-containing genes. The relative small R^2 ^value between gene expression variation and toxicity degree alone may be explained by the absence of association between them within the non-TATA-containing genes.

Previous studies identified the number of *cis*-element motifs contributing to gene expression variation in *A. thaliana *[3], but Landry et al. [6] found that the number of *cis*-elements only marginally affects gene expression variation in yeast. Here we studied these two factors (the number of *cis*-elements and the number of TFs) in relation to gene expression variation. We first analyzed the relationship between gene expression variation and number of *cis*-elements [26] using the linear model. Similar to MacIsaac et al. [26], we defined *cis*-elements according to binding probability (p < 0.0001) and classified them according to their conservation in two other yeast species. The results are presented in Table 1 for the different gene expression data. For the Ca and Na exposure data [19], the highest R^2 ^is 1.33% (p-value = 4.45e-08) when *cis*-elements that are conserved in at least one species are used. We then studied the relationship between gene expression variation and the number of TFs that influence target gene expression based on the gene regulatory network developed in Hu et al. [27]. A highly significant positive correlation between gene expression variation and the number of TFs was observed (R^2 ^= 8.28%, β = 0.0654, p-value < 2.2e-16) (Figure 3B). The fraction of variation explained by the number of TFs (R^2 ^= 8.28%) is much higher than that by the number of *cis*-elements indicating that the number of TFs is a better predictor of gene expression variation than the number of *cis*-elements. This result implies the importance of trans-effect for gene expression variation.

**Table 1:** The relationship between gene expression variation and the number of *cis*-elements.

*Cis-elements are identified with binding p < 0.0001 and conservation in at least 2 other yeast species*			
Gene expression data-set	Linear regression		R^2^
		
	β	p value	

Ca_Na exposure	0.0589	1.17e-06	1.25%
Chemostat	0.0188	0.0067	0.39%
Environmental Stress	0.0507	5.48e-07	1.33%
Oxidative Stress	0.0025	0.725	0.01%

*Cis-elements are identified with binding p < 0.0001 and conservation in at least 1 other yeast*			

Gene expression data-set	Linear regression		R^2^
		
	β	p value	

Ca_Na exposure	0.0539	4.45e-08	1.33%
Chemostat	0.0161	0.0046	0.36%
Environmental Stress	0.0516	3.56e-10	1.74%
Oxidative Stress	0.0013	0.826	0

*Cis-elements are identified with binding p < 0.0001 and no Conservation Criteria*			

Gene expression data-set	Linear regression		R^2^
		
	β	p value	

Ca_Na exposure	0.0393	1.05e-07	1.02%
Chemostat	0.0151	0.0004	0.45%
Environmental Stress	0.0346	1.68e-08	1.15%
Oxidative Stress	0.0028	0.526	0.01%

*Cis*-elements are identified with three different criteria according to their conservation in two other species. β is the linear coefficient in the linear model, the p-value is related to the null hypothesis that β ≠ **0 **versus β = **0**, and the R^2 ^is the fraction of variation explained by the number of *cis*-elements.

### Overall analysis of factors affecting gene expression variation

As enumerated above, we have identified several factors influencing gene expression variation in *S. cerevisiae*. In addition to the presence/absence of TATA box identified in previous studies [6,7], we found that gene expression variation decreases as both the protein interaction and toxicity modulation degrees increase. These findings are consistent with other studies for expression variation of single cells [9] and evolutionary expression variation across different strains/species [4,5,25]. We also found that gene expression variation increases as the number of TFs or the number of *cis*-elements increases and that the number of TFs regulating a gene is a much better predictor of expression variation than the number of *cis*-elements. We therefore studied the contribution of each factor and their interactions on gene expression variation by taking the other factors into consideration using the Akaike Information Criterion (AIC) [28]. We retained the model with the smallest AIC. Table 2 gives the results of factors included in the final linear model using the MIPS interaction data [12] and the four expression profiles by stepwise selection with AIC. We then studied the effect of the selected factors on expression variation using linear regression, and the corresponding p-values and R^2 ^values are given in Table 2. The results showed that protein interaction degree only explained less than 1% of variation when adjusted for the three other main factors and two interaction terms. Except for the oxidative stress gene expression dataset [22], the consistently selected model contained four main factors including protein interaction degree, toxicity degree, the number of TFs, and TATA box, and two interaction terms, i.e., interaction between TATA and toxicity degree and TATA and number of TFs. The two interaction terms are found to be statistically significant across three expression datasets: Ca and Na exposure [19], chemostat [20], and environmental stress [21] (p-value < 0.05) and explained 0.4% – 2.7% of the variation.

**Table 2:** Analysis of four factors and their interactions affecting expression variation using stepwise selection with AIC.

variable	Ca_Na_exposure			Chemostat			Environmental Stress			Oxidative Stress		
	model	p value	R^2^	model	p value	R^2^	model	p value	R^2^	model	p value	R^2^

x1	√	0.4252	0.05%	√	0.1582	0.16%	√	0.0647	0.28%	√	0.1075	0.21%
x2	√	0.3721	0.06%	√	0.0635	0.28%	√	0.0861	0.24%		0.8729	0.002%
x3	√	1.16E-20	6.76%	√	4.59E-09	2.73%	√	< 2e-16	13.42%	√	7.30E-09	2.65%
x4	√	3.22E-12	3.83%	√	7.96E-10	3.00%	√	< 2e-16	6.92%	√	0.8841	0.002%
x1*x2												
x1*x3												
x1*x4	√	0.0581	0.29%	√	0.0100	0.53%				√	0.0348	0.36%
x2*x3				√	0.0422	0.33%						
x2*x4	√	0.0226	0.41%	√	0.0036	0.68%	√	0.0108	0.53%			
x3*x4	√	0.0039	0.67%	√	0.0185	0.45%	√	6.2e-09	2.71%			
R^2^model	16.36%			12.73%			22.39%			4.43%		

The four main factors include protein interaction degree (x1), toxicity degree (x2: treat essential genes as ones with toxicity degree 4), number of TFs (x3), and the presence of TATA box (x4: 1-TATA containing genes, 0-non-TATA containing genes). The protein interaction data used in this analysis is based on the MIPS dataset. The column marked with "√" indicates inclusion in the final linear model. The multiple linear regression is based on the final linear model, respectively. The p-value is related to the null hypothesis that β ≠ **0 **versus β = **0**. R^2 ^is the variation explained by the model and each independent variable, respectively.

Because we found that TATA box interact with toxicity degree and the number of TFs influencing gene expression variation, we reanalyzed the contributions of toxicity degree and number of TFs on gene expression variation stratified by the presence/absence of TATA box using the linear model. The protein physical interaction degree was not included in this analysis because the interaction between TATA box and physical interaction degree was not detected. Table 3 shows the different results for TATA and non-TATA gene sets. First, the number of TFs is the most significant factor for gene expression variation in both TATA- and non-TATA-containing genes with p-values less than 5e-6 for all the datasets. However, the association strength between the number of TFs and gene expression variation measured by the coefficient β for the non-TATA-containing genes is about 1.5-fold higher than the corresponding values for the TATA-containing genes. Accordingly, the p-values for the non-TATA-containing genes are about three orders of magnitude smaller than the corresponding p-values for the TATA-containing gene set. We noted that TATA-containing genes tend to have higher number of TFs than non-TATA-containing genes (p-value = 1.184e-10), but this cannot explain our observation that the association strength between expression variation and the number of TFs within the TATA-containing genes is lower than that within the non-TATA-containing genes. One possible explanation is that the presence of TATA-box weakens the effect of the number of TFs on gene expression during evolution. Second, the toxicity degree is only a significant contributor for gene expression variation within the TATA-containing genes in the Ca-Na exposure [19] and Chemostat [20] gene expression dataset, while it is not a significant contributor for gene expression variation in the non-TATA-containing gene set. Within the environmental stress gene expression dataset [21], the effect of toxicity degree on gene expression variation was not statistically significant. However, we did observe a decreasing trend of gene expression variation with respect to toxicity degree within the TATA-containing genes (see Additional File 1, Supplemental Figure 5).

**Table 3:** The effect of two factors on expression variation stratified by the presence/absence of TATA box.

TATA dataset												
variable	Ca_Na_exposure			Chemostat			Environmental Stress			Oxidative Stress		
	
	β	p-value	R^2^	β	p-value	R^2^	β	p-value	R^2^	β	p-value	R^2^

x2	-0.1436	0.0024	1.95%	-0.0764	0.0034	1.81%	-0.0510	0.156	0.43%	0.0081	0.778	0.02%
x3	0.0312	5.4e-10	7.90%	0.0168	1.5e-09	7.50%	0.0283	2.6e-13	10.79%	0.0138	4.8e-06	4.37%
R^2 ^(model)	9.47%			8.97%			11.09%			4.39%		

Non-TATA dataset												

variable	Ca_Na_exposure			Chemostat			Environmental Stress			Oxidative Stress		
	
	β	p-value	R^2^	β	p-value	R^2^	β	p-value	R^2^	β	p-value	R^2^

x2	-0.0167	0.402	0.06%	-0.0026	0.8189	0.005%	0.0258	0.086	0.26%	0.0038	0.747	0.009%
x3	0.0502	< 2e-16	8.70%	0.0300	< 2e-16	9.56%	0.0468	< 2e-16	12.6%	0.0181	4.4e-10	3.40%
R^2 ^(model)	8.85%			9.62%			12.67%			3.39%		

The linear model that includes the toxicity degree (x2) and the number of TFs (x3) is built for each of the four gene expression datasets, respectively. R^2 ^is the variation explained by each independent factor and the model, respectively. β is the linear coefficient in the linear model, and the p-value is related to the null hypothesis that β ≠ **0 **versus β = **0**.

The toxicity degree of a gene measures the tolerance of the yeast cell to different external stress conditions when the gene is knocked out. Therefore, it might be expected that the different relationship between gene expression variation and toxicity degree for TATA-containing genes and non-TATA-containing genes is due to the enrichment of stress-related genes in TATA-containing genes, as found in [17]. To test this hypothesis, we used a set of genes related to Environmental Stress Response (ESR) [21]. If the hypothesis is true, we would expect a higher association between expression variation and toxicity degree within the ESR genes than that within the non-ESR genes. However, our data shows that the β values are similar for the two groups of genes (Table 4). Thus we cannot explain the interaction between TATA and toxicity degree by the enrichment of ESR genes in TATA-containing genes. The biological mechanisms underlying the observed interaction are not clear and need to be further studied.

**Table 4:** The effect of toxicity degree on expression variation stratified by the set of environmental stress response (ESR).

Gene group	Ca_Na_exposure		Chemostat		Environmental Stress		Oxidative Stress	
	
	β	p-value	β	p-value	β	p-value	β	p-value
ESR	-0.0710	0.0053	-0.0314	0.0532	-0.0176	0.3640	0.0026	0.8708
Non-ESR	-0.0848	2.09e-13	-0.0354	1.11e-07	-0.0579	1.28e-11	-0.0091	0.2086

The linear model is built for each of the four gene expression datasets, respectively. β is the linear coefficient in the linear model and the p-value is related to the null hypothesis that β ≠ **0 **versus β = **0**.

We also did the same analysis for the average gene expression variation across the four expression datasets (Ca and Na exposure [19], chemostat [20], environmental stress [21], and oxidative stress [22]) and the combined gene expression data of Landry et al. [7], and the results are presented as Additional File 2. The same conclusions can be obtained indicating the robustness of our results. Previous studies showed that TATA- and non-TATA-containing genes might recruit different coactivator complexes for gene expression [17]. TATA-containing genes were also found to be subject to greater nucleosomal regulation than non-TATA-containing genes [17]. Basehoar et al. [17] suggested that two distinct regulatory mechanisms may be present at TATA- and TATA-less promoters. The results in Table 3 support their findings.

The results based on the oxidative stress gene expression dataset [22] are not consistent with the results based on the other three gene expression datasets. This observation may be due to the relatively small gene expression variation in this data. For example, the range of the variance of the expression levels within the oxidative stress dataset, (0.07, 5.34), is much smaller than the corresponding ranges, (0.02, 10.59), (0.17, 9.18), and (0.09, 11.07), for the Ca and Na exposure [19], chemostat [20], and environmental stress conditions [21], respectively.

We also studied the contributing factors for gene expression variation using the DIP [13] and BioGrid interactions [14], and the results are given in Additional File 1 and File 2. Similar conclusions as those based on the MIPS interaction data [12] were obtained. The consistency of the results using different combinations of protein interaction data sets and gene expression profiles showed the robustness of our conclusions. However, the fraction of gene expression variation explained by all factors is less than 25%. One possible explanation is that the measurement of gene expression changes and other factors, including the toxicity degree and interaction degree, are still very noisy. We expect that the true R^2 ^would be higher than that observed in this study.

## Conclusion

We implemented a system-wide analysis of proteomic and genomic factors affecting gene expression variation. Among four different factors (protein interaction degree, toxicity degree, TATA box, the number of TFs), TATA-box and the number of TFs are the most important factors influencing gene expression variation. The influence of TATA-box on evolutionary gene expression variation has been extensively studied both computationally and experimentally [6,8], and our results are consistent with their findings. Although it is intuitive that the number of TFs regulating a gene should have a significant effect on the gene's expression variation, the magnitude of its influence has not been studied in large scale expression datasets to the best of our knowledge. Our findings demonstrated that the gene regulation is a main factor affecting gene expression variation. Protein interaction degree and toxicity degree do not account for as much variation when compared to the influence of the number of TFs and the TATA-box.

In our overall analysis, we also found the interactions between TATA-box and toxicity degree as well as the number of TFs influence expression variation. The further study stratified by TATA-box indicated that TATA-containing genes and non-TATA containing genes behave differently in relation to the toxicity degree and the number of TFs. The effect of the number of TFs on expression variation within the TATA-containing genes is lower than that for the non-TATA-containing genes. On the other hand, toxicity degree is associated with expression variation within the TATA-containing genes only. These findings suggest that the regulatory mechanism might be more complicated for TATA-containing genes than non-TATA containing genes.

## Methods

In order to study factors affecting gene expression variation, we collected data on gene expression profiles, protein physical interactions, gene regulatory networks, essentiality and toxicity resistance. Details of these data are given below.

### Gene expression profiles

A large number of gene expression studies are available. In this study, we chose gene expression studies containing at least 40 conditions. These datasets include yeast gene expression profiles under 40 Ca and Na exposure conditions [19], chemostat (i.e., nutritional stress) at 100 conditions [20], environmental stress at 156 conditions [21] and oxidative stress at 70 conditions [22]. These data were analyzed separately to ensure that between-laboratory variation was minimized. A combined gene expression profile under more than 1,500 conditions was collected by [7]. The responsiveness for each gene across more than 1,500 conditions calculated by [7] was used in our analysis as expression variation.

### Protein interaction data

We downloaded yeast protein interaction data from three different data sources. The MIPS (Munich Information Center for Protein Sequences) [12] dataset (version: PPI_18052006.tab) contains 11,124 protein physical interactions involving 4,404 proteins. The DIP core interaction dataset [13] (version: ScereCR20070107) contains 5,738 protein interactions involving 2,161 proteins. The DIP core interactions were assessed by a number of quality tests and are supposed to be highly reliable [11]. The BioGrid [14] dataset (version 2.0.34) contains 59,317 protein physical interactions involving 5,054 proteins.

### Essential and toxicity modulating genes

Large scale gene deletion studies have identified about 17–20% of the genes essential for yeast cell survival [15] under normal conditions. Even within the class of non-essential genes, a gene's importance in relation to cell survival is not the same. Further studies classified the non-essential genes based on the cell's fitness phenotypes under four different DNA damage perturbations when a gene is knocked out [16]. The toxicity modulating genes were defined as those significantly affecting the cell's fitness phenotype when knocked out. We defined the toxicity degree of a gene as the number of perturbations that significantly affected the deletion strain's fitness. Essential genes were downloaded from the SGD website [29], and the toxicity degrees of non-essential genes were calculated from [16].

### Gene Regulatory Network

Studies have shown that gene expression variation is positively correlated with the number of *cis*-regulatory elements and the length of intergenic region in several organisms. Since *cis*-elements control the expression of genes through interaction with the TFs, it is interesting to study if the number of TFs regulating a gene has an effect on gene expression variation. The mapping of *cis*-elements to genes was obtained using motif discovery algorithms, PhyloCon and Converge, with binding p-value less than 0.001 and conservation in at least 0, 1 or 2 other yeast species [26]. The mapping of the TFs to genes is obtained from Hu et al. [27].

### TATA-containing genes

A TATA box is a DNA sequence (*cis*-element) found in the promoter region of most eukaryotic genes. The TATA consensus sequence was identified as TATA(A/T)A(A/T)(A/G) [17]. The TATA box has been identified as a very important factor for gene expression variation. The relationship between yeast genes and the TATA box was downloaded from [17]. There are 1090 out of 6278 genes that were predicted to have a TATA box. Our analysis used these 1090 genes as TATA-containing genes and other genes as non-TATA-containing genes. (We note that 607 genes are not classified in [17], and the results are essentially the same when these genes are not considered (data not shown).)

### Statistical Analysis

Gene expression variation was measured by the logarithm of the variance of the gene expression levels under various conditions. The distribution of the variance was not normal. In addition, the standard deviations of the resulting distributions conditional on the independent variables (protein physical interaction degree, toxicity degree, TATA box, number of TFs) differed widely, making the linear model for the variance invalid. To avoid these problems, we measured the gene expression variation by the logarithm of the variance. The resulting distributions seem to fit the conditions for the linear model. Hence, in our study, we used a linear model to study the relationship between the expression variation and each factor. In the study of the relationship between the gene expression variation and interaction degrees, we first used the LOWESS function in R [23] to fit the data. An approximate linear relationship between gene expression variation and interaction degree was observed when the interaction degree was less than 20. We then proceeded to use linear regression to fit the data up to interaction degree 20.

*v *= *α *+ *βd*

where *v *is the gene expression variation and *d *is the interaction degree. α and β are parameters. We tested the statistical significance for the relationship between gene expression variation and interaction degree based on the linear regression model.

Before we do the joint analysis of expression variation with respect to the four factors (protein interaction degree, toxicity degree, number of TFs, and TATA box), we tested if the four factors are highly correlated. We calculated the correlation matrix between them and it is given in Additional File 2 (Supplementary Table 9). All the correlation coefficients are smaller than 0.3 indicating that they are not highly correlated. Although it might be more computationally reasonable to first find the principal components of these factors and then analyze the data using linear regression, the interpretation of the final result is not clear. Since these factors are not highly correlated, we treat them as independent factors in our joint analysis.

In the overall analysis, we first used stepwise selection to find a model that gives the smallest AIC (Akaike information criterion) = 2*K+n*ln*(SSE/n)*, where *K *is the number of parameters in the model; *n *is the number of observations; and SSE is the residual sum of squares [30]. We then used linear regression to analyze the relationship between gene expression variation and the retained factors and interactions. The corresponding p-values and the R^2 ^values are reported in Table 2.

## Authors' contributions

All authors contributed to the design and coordination of the study. LZ carried out the calculations and performed the statistical analysis. FS drafted the original manuscript, which was revised by XM and LZ. All authors read and approved the final manuscript.

## Supplementary Material

Additional file 1 The statistical analysis of individual factors on gene expression variation using four expression datasets, respectively. Click here for file

Additional file 2 Overall analysis of four main factors (protein interaction degree, toxicity degree, number of TFs and the presence of TATA box) and their interactions. Click here for file
