# Supplementary material for: The effects of protein interactions, gene essentiality and regulatory regions on expression variation
Source: BMC Syst Biol. 2008 Jun 26;2:54. doi: 10.1186/1752-0509-2-54 (PMC2474594; doi:10.1186/1752-0509-2-54)
Supplement: Additional file 1 — The statistical analysis of individual factors on gene expression variation using four expression datasets, respectively. [file 1752-0509-2-54-S1.pdf]

Table of Contents

1. Figures using the MIPS Interaction Data Set ………………………..2

2. Figures Using the DIP Interaction Data Set ……………………….…...11

3. Figures Using the BioGrid Interaction Data Set ……………………19

Supplementary Figures using the MIPS Interaction Data Set

Chemostat (Nutritional Stress)

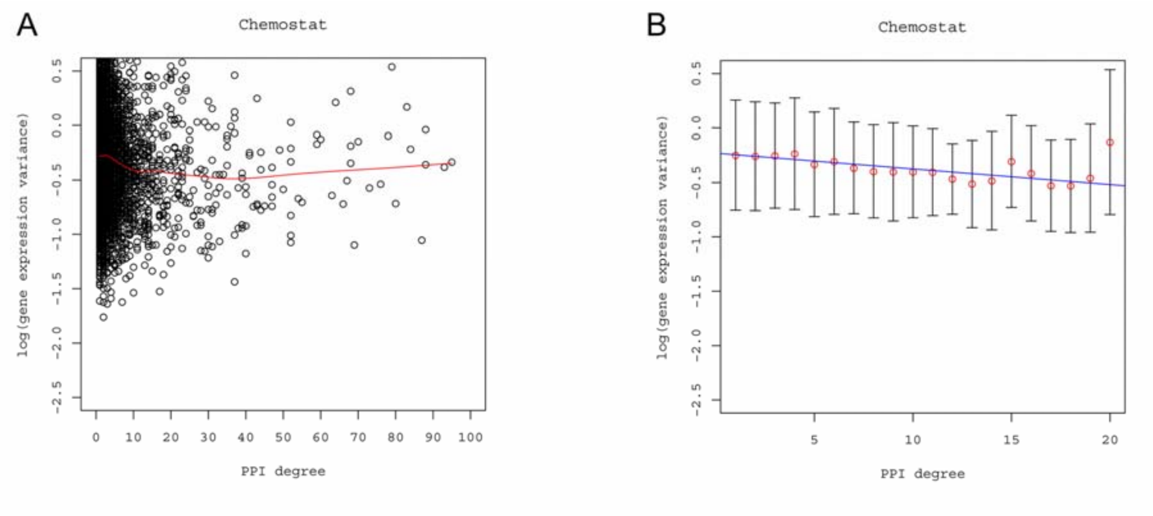

Supplemental Figure 1. Gene expression variation is negatively correlated with protein interaction degree. The x-axis represents protein physical interaction degree, and the y-axis represents gene expression variation. A) The LOWESS fit to the gene expression variation. B) Bar-plot of the expression variation of all the genes with a given protein interaction degree together with the linear regression fit to the gene expression variation in relation to the interaction degree. The linear coefficient $\beta = -0.0145$, $R^2 = 1.05\%$, and the p-value = 1.279e-10. The red dots are the mean expression variation of the genes given the protein physical interaction (PPI) degree. The bar represents the standard deviation of gene expression variation given PPI degree.

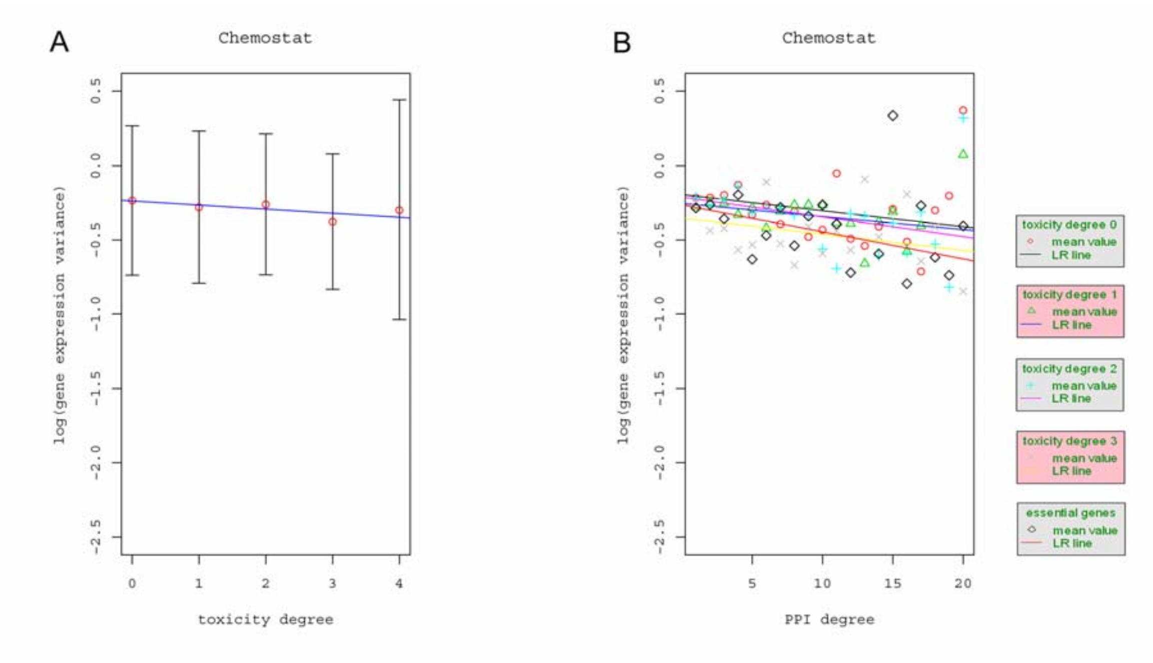

Supplemental Figure 2. The effect of essentiality, toxicity degree, and protein interaction degree on gene expression variation. A) Bar-plot of the expression variation of all the genes with a given toxicity degree together with the linear regression fit to the expression variation of the genes in relation to the toxicity degree. The linear coefficient β = -0.0275, R^2 = 0.46%, and the p-value = 1.632e-05. B) The mean expression variation and the linear regression fit to the expression variation with respect to PPI degree for non-essential genes stratified according to toxicity degree and for the essential genes The β values are -0.0108, -0.0089,-0.0133,-0.0109, and -0.0183 for toxicity degree 0, 1, 2, and 3, and the essential genes, respectively. The corresponding p-values are 0.0120, 0.0914, 0.0113, 0.1355, and 0.0163, respectively. R^2 is 0.42%, 0.36%, 1.65%, 1.2% and 2.47%, respectively. The labels are the same as those in Supplemental Figure 1.

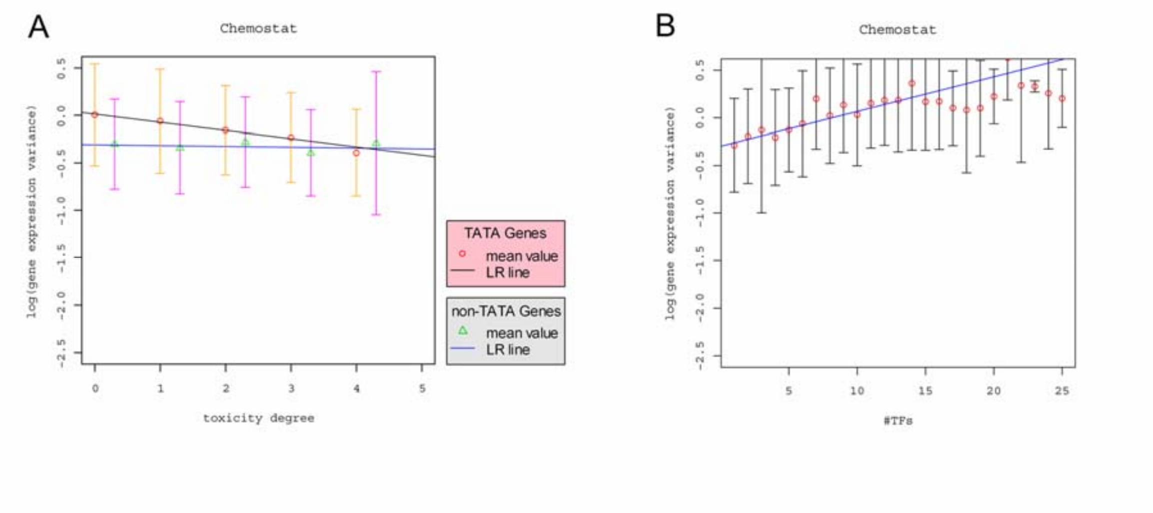

Supplemental Figure 3. The effect of TATA box, number of TFs, and toxicity degree on gene expression variation. A) The relationship between expression variation and toxicity degree stratified by the presence/absence of the TATA box (R^2 = 2.33%, β = -0.0860, p-value = 2.104e-05 for TATA containing group; R^2 = 0.05%, β = -0.0085, p-value = 0.1906 for the non-TATA containing group); B) The relationship between expression variation and the number of TFs up to 25 (R^2 = 8.16%, β = 0.0364, p-value < 2.2e-16). The labels are the same as those in Supplemental Figure 1.

Environmental Stress

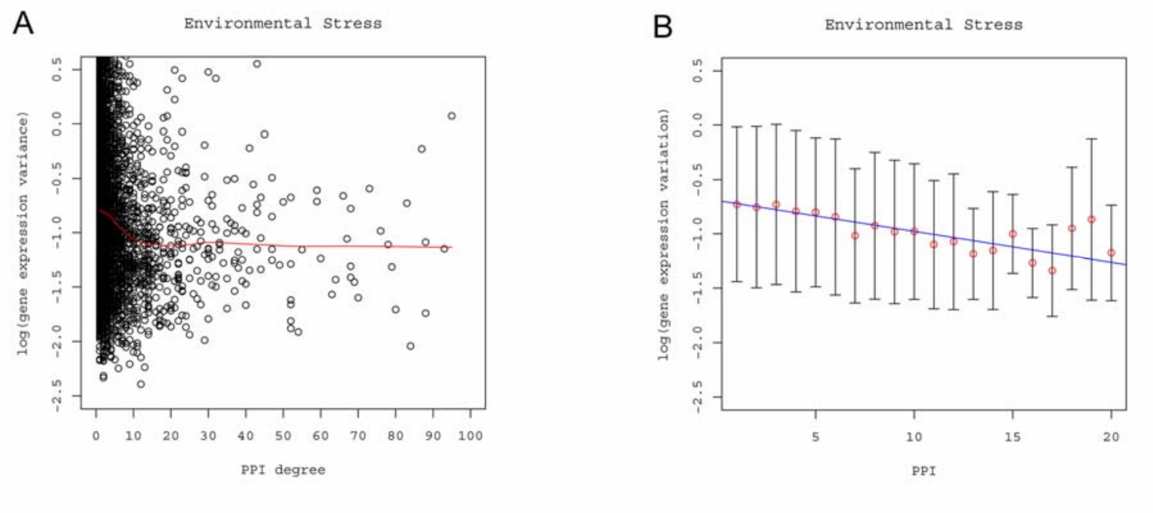

Supplemental Figure 4. Gene expression variation is negatively correlated with protein interaction degree. The x-axis represents protein physical interaction degree, and the y-axis represents gene expression variation. A) The LOWESS fit to the gene expression variation. B) Bar-plot of the expression variation of all the genes with a given protein interaction degree together with the linear regression fit to the gene expression variation in relation to the interaction degree. The linear coefficient $\beta = -0.0286$, $R^2 = 1.96\%$, and the p-value = 2.2e-16. The red dots are the mean expression variation of the genes given the protein physical interaction (PPI) degree. The bar represents the standard deviation of gene expression variation given PPI degree.

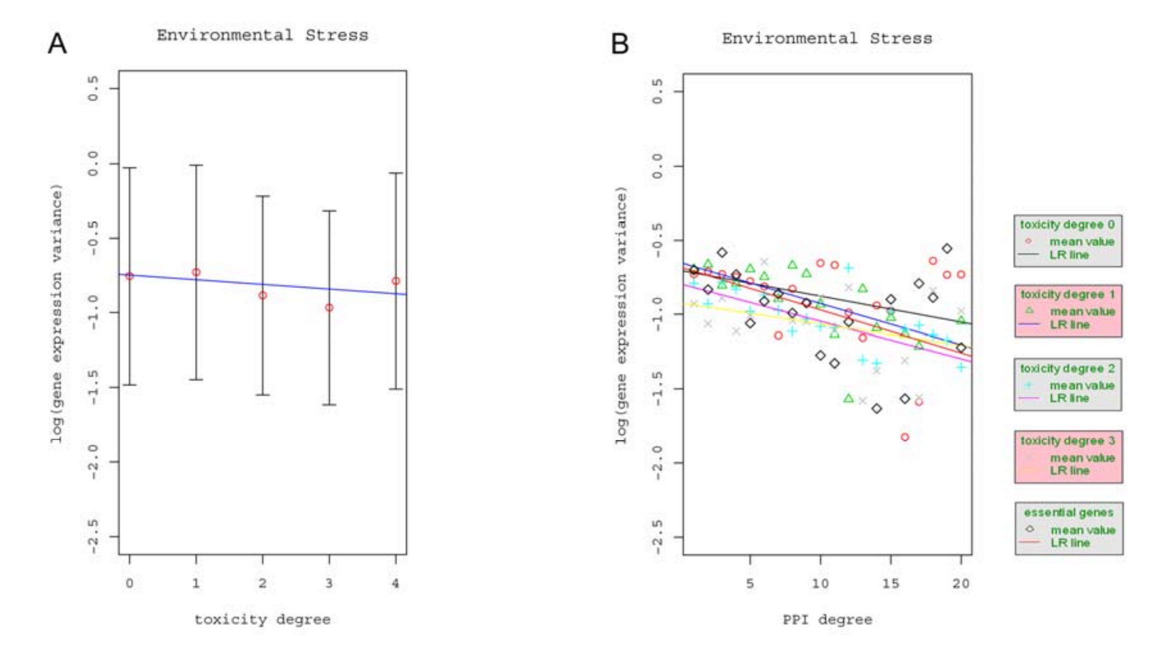

Supplemental Figure 5. The effect of essentiality, toxicity degree, and protein interaction degree on gene expression variation. A) Bar-plot of the expression variation of all the genes with a given toxicity degree together with the linear regression fit to the expression variation of the genes in relation to the toxicity degree. The linear coefficient β= -0.0314, R^2 = 0.3%, and the p-value = 0.0006. B) The mean expression variation and the linear regression fit to the expression variation with respect to PPI degree for non-essential genes stratified according to toxicity degree and for the essential genes. The β values are -0.0175, -0.0277, -0.0253, -0.0143, and -0.0289 for toxicity degree 0, 1, 2, and 3, and the essential genes, respectively. The corresponding p-values are 0.0049, 0.0002, 0.0007, 0.1553, and 0.0162, respectively. R^2 is 0.53%, 1.7%, 2.93%, 1.09% and 2.48%, respectively. The labels are the same as those in Supplemental Figure 4.

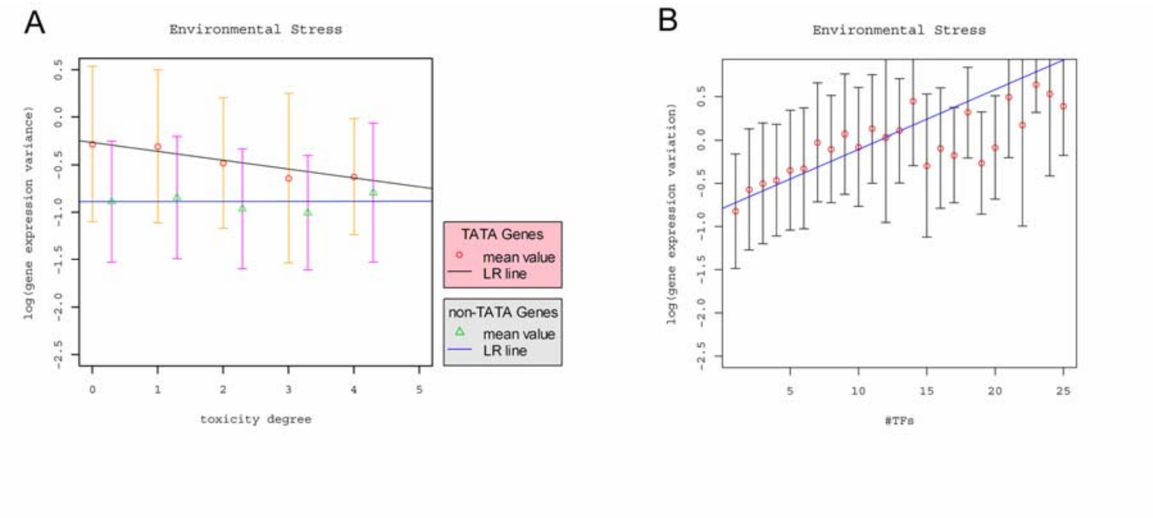

Supplemental Figure 6. The effect of TATA box, number of TFs, and toxicity degree on gene expression variation. A) The relationship between expression variation and toxicity degree stratified by the presence/absence of the TATA box (R^2 = 1.23%, β=-0.0936, p-value = 0.0020 for TATA containing group; R^2 = 0, β= 0.0007, p-value = 0.9329 for the non-TATA containing group); B) The relationship between expression variation and the number of TFs up to 25 (R^2 = 14.22%, β=0.0687, p-value < 2.2e-16). The labels are the same as those in Supplemental Figure 4.

Oxidative Stress

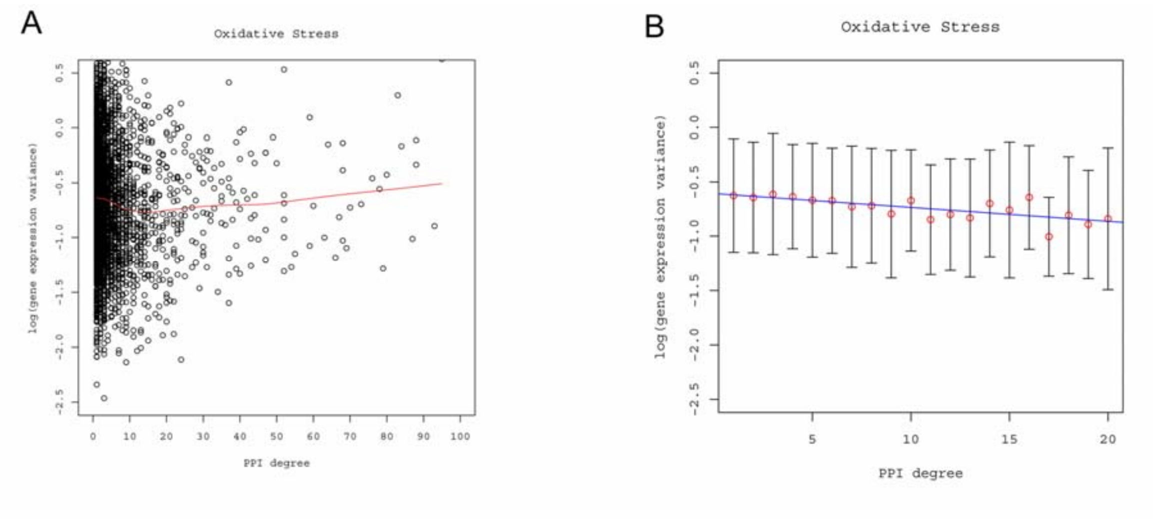

Supplemental Figure 7. Gene expression variation is negatively correlated with protein interaction degree. The x-axis represents protein physical interaction degree, and the y-axis represents gene expression variation. A) The LOWESS fit to the gene expression variation. B) Bar-plot of the expression variation of all the genes with a given protein interaction degree together with the linear regression fit the gene expression variation in relation to the interaction degree. The linear coefficient $\beta = -0.0128$, $R^2 = 0.72\%$, and the p-value = 9.543e-08. The red dots are the mean of expression variation of the genes given the protein physical interaction (PPI) degree. The bar represents the standard deviation of the gene expression variation given PPI degree.

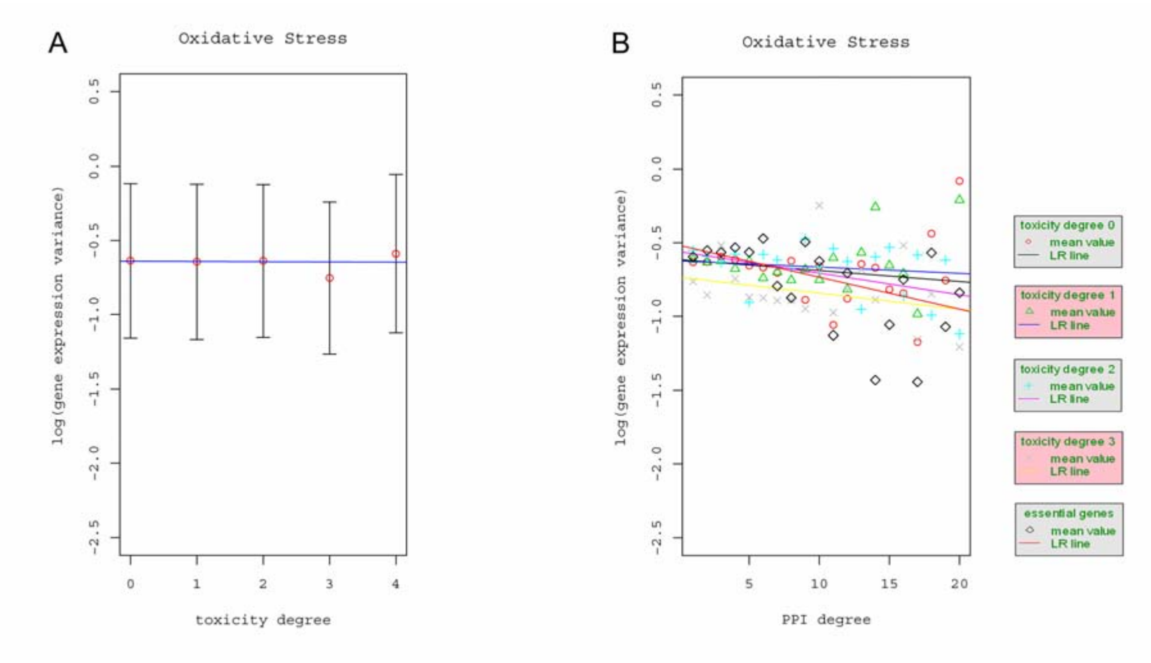

Supplemental Figure 8. The effect of essentiality, toxicity degree, and protein interaction degree on gene expression variation. A) Bar-plot of the expression variation of all the genes with a given toxicity degree together with the linear regression fit to the expression variation of the genes in relation to the toxicity degree. The linear coefficient β= -0.0016, R^2 = 0 and the p-value = 0.8162. B) The mean expression variation and the linear regression fit to the expression variation with respect to PPI degree for non-essential genes stratified according to toxicity degree and for the essential genes. The β values are -0.0075, -0.0043, -0.0147, -0.0109, and -0.0217 for toxicity degree 0, 1, 2, and 3, and the essential genes, respectively. The corresponding p-values are 0.0942, 0.4425, 0.0114, 0.1821, and 0.0186, respectively. R^2 is 0.19%, 0.07%, 1.65%, 0.96% and 2.38%, respectively. The labels are the same as those in Supplemental Figure 7.

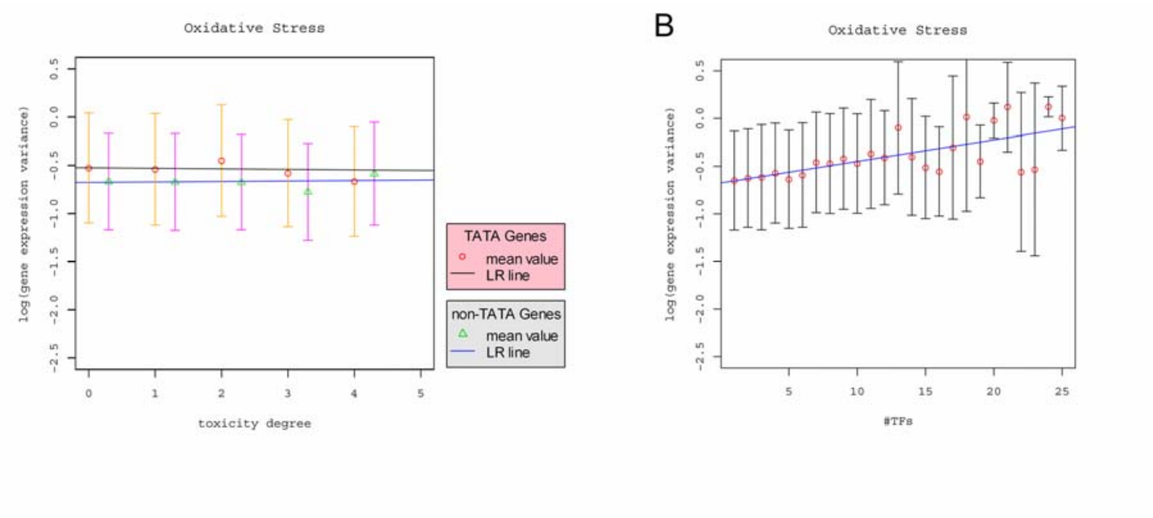

Supplemental Figure 9. The effect of TATA box, number of TFs, and toxicity degree on gene expression variation. A) The relationship between expression variation and toxicity degree stratified by the presence/absence of the TATA box (R^2 = 0.01%, β= -0.0054, p-value = 0.8048 for TATA containing group; R^2 = 0.02%, β= 0.0050, p-value = 0.4737 for the non-TATA containing group); B) The relationship between expression variation and the number of TFs up to 25 (R^2 = 2.97%, β=0.0226, p-value < 2.2e-16). The labels are the same as those in Supplemental Figure 7.

Supplementary Figures Using the DIP Interaction Data Set

Ca and Na exposure

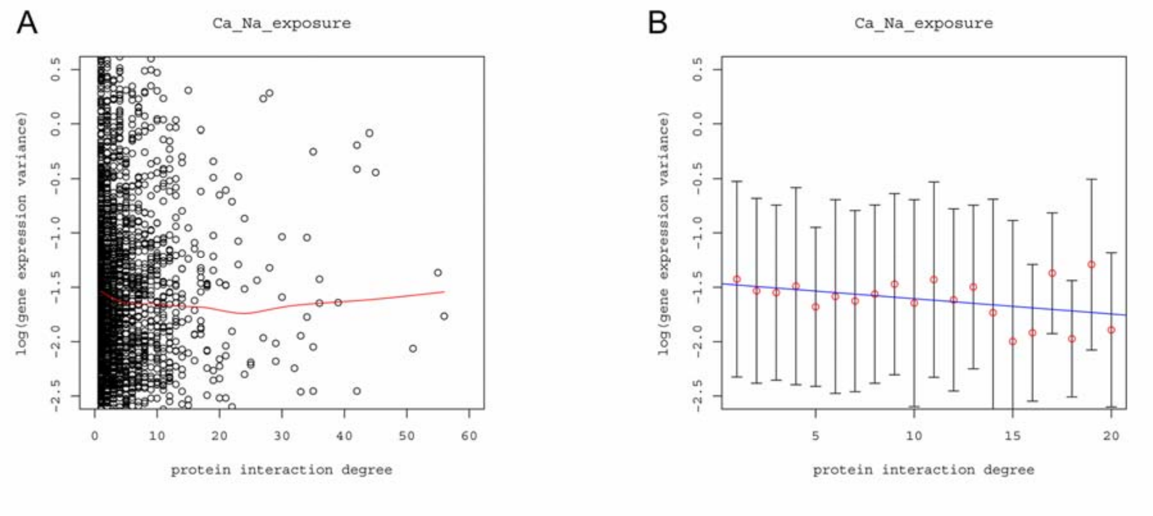

Supplemental Figure 10. Gene expression variation is negatively correlated with protein interaction degree. The x-axis represents protein physical interaction degree, and the y-axis represents gene expression variation. A) The LOWESS fit to the gene expression variation. B) Bar-plot of the expression variation of all the genes with a given protein interaction degree together with the linear regression fit to the gene expression variation in relation to the interaction degree. The linear coefficient $\beta = -0.0140$, $R^2 = 0.39\%$, and the p-value = 0.0041. The red dots are the mean expression variation of the genes given the protein physical interaction (PPI) degree. The bar represents the standard deviation of gene expression variation given PPI degree.

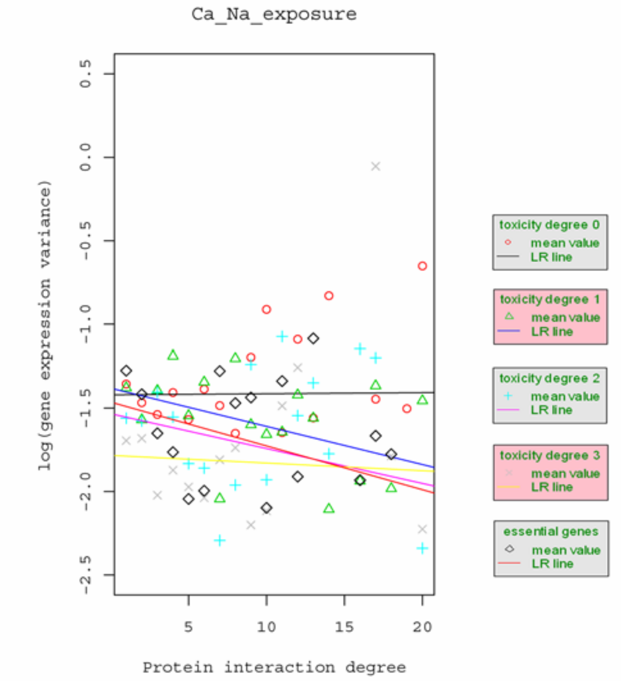

Supplemental Figure 11. The effect of toxicity degree, and protein interaction degree on gene expression variation. The mean expression variation and the linear regression fit to the expression variation of all the genes with respect to protein interaction degree for non-essential genes stratified according to toxicity degree and for the essential genes. The β values are 0.0007, -0.0229, -0.0209, -0.0046, and -0.0263 for toxicity degree 0, 1, 2, and 3, and the essential genes, respectively. The corresponding p-values are 0.9555, 0.0547, 0.1321, 0.7783, and 0.1085, respectively. R^2 is 0, 0.95%, 0.96%, 0.06% and 1.37%, respectively. The labels are the same as those in Supplemental Figure 10.

Chemostat (nutritional stress)

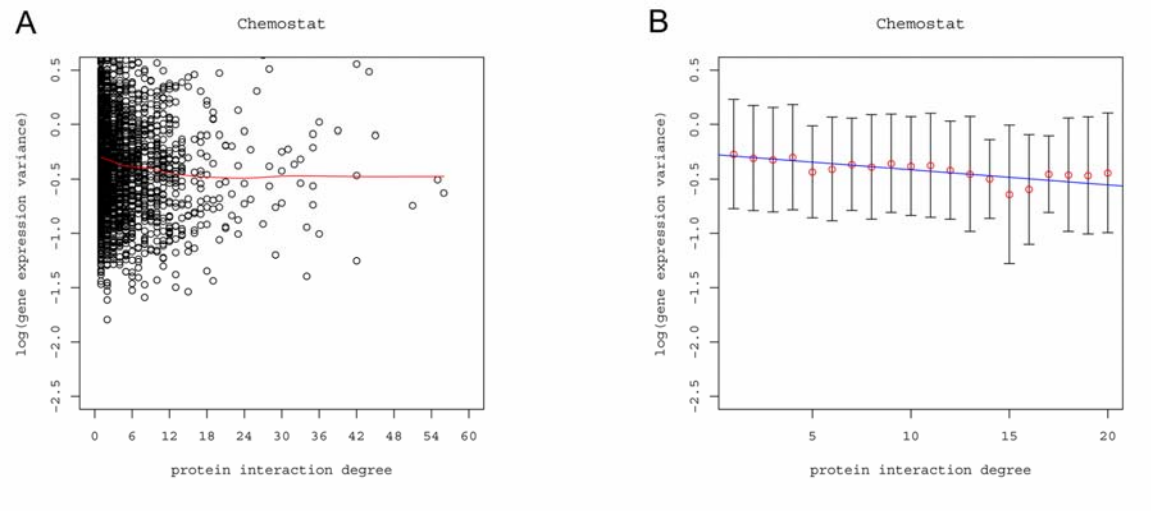

Supplemental Figure 12. Gene expression variation is negatively correlated with protein interaction degree. The x-axis represents protein physical interaction degree, and the y-axis represents gene expression variation. A) The LOWESS fit to the gene expression variation. B) Bar-plot of the expression variation of all the genes with a given protein interaction degree together with the linear regression fit to the gene expression variation in relation to the interaction degree. The linear coefficient $\beta = -0.0140$, $R^2 = 1.25\%$, and the p-value = 2.458e-07. The red dots are the mean expression variation of the genes given the protein physical interaction (PPI) degree. The bar represents the standard deviation of gene expression variation given PPI degree.

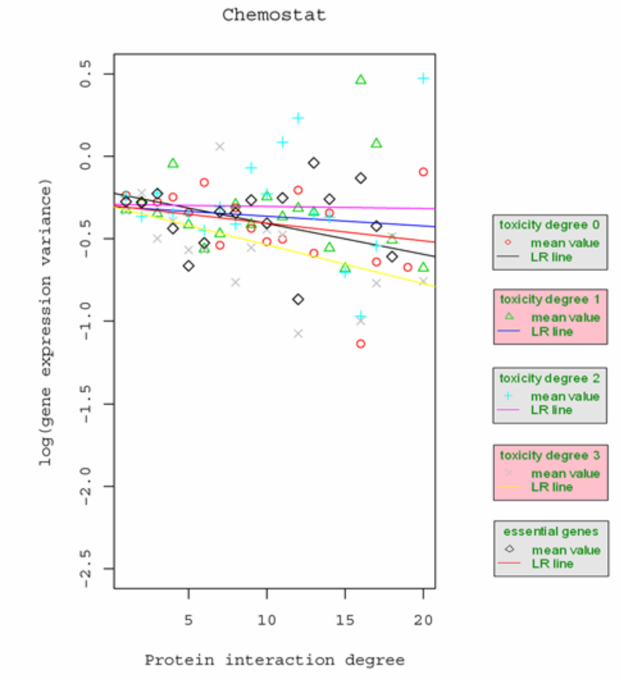

Supplemental Figure 13. The effect of toxicity degree, and protein interaction degree on gene expression variation. The mean expression variation and the linear regression fit to the expression variation of all the genes with respect to protein interaction degree for non-essential genes stratified according to toxicity degree and for the essential genes. The β values are -0.0187, -0.0060, -0.0013, -0.0235, and -0.0108 for toxicity degree 0, 1, 2, and 3, and the essential genes, respectively. The corresponding p-values are 0.0045, 0.3927, 0.8726, 0.0173, and 0.1775, respectively. R^2 is 1.38%, 0.19%, 0.01%, 4% and 0.97%, respectively. The labels are the same as those in Supplemental Figure 12.

Environmental Stress

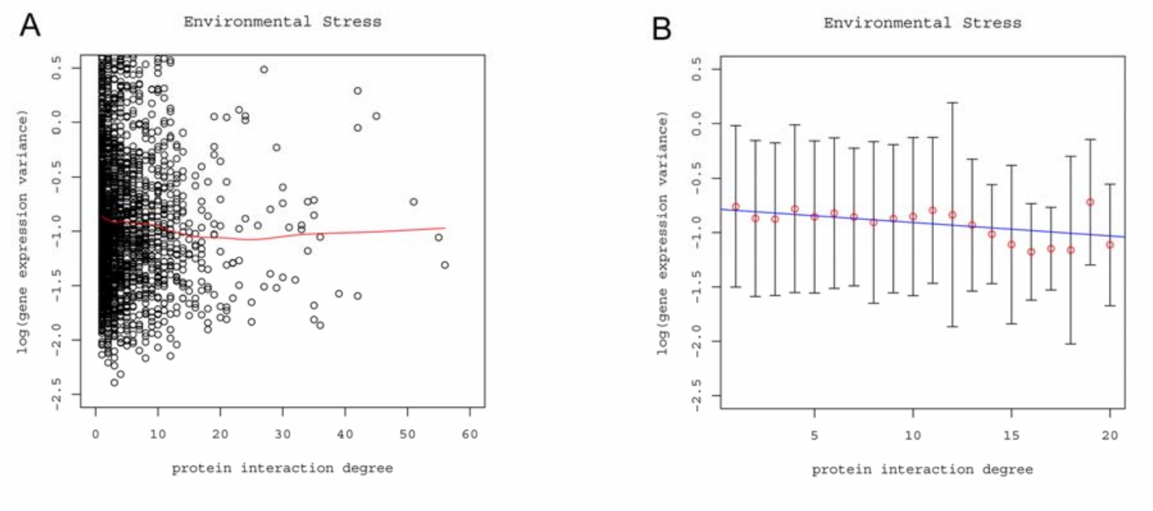

Supplemental Figure 14. Gene expression variation is negatively correlated with protein interaction degree. The x-axis represents protein physical interaction degree, and the y-axis represents gene expression variation. A) The LOWESS fit to the gene expression variation. B) Bar-plot of the expression variation of all the genes with a given protein interaction degree together with the linear regression fit to the gene expression variation in relation to the interaction degree. The linear coefficient $\beta = -0.0124$, $R^2 = 0.45\%$ and the p-value = 0.0023. The red dots are the mean expression variation of the genes given the protein physical interaction (PPI) degree. The bar represents the standard deviation of gene expression variation given PPI degree.

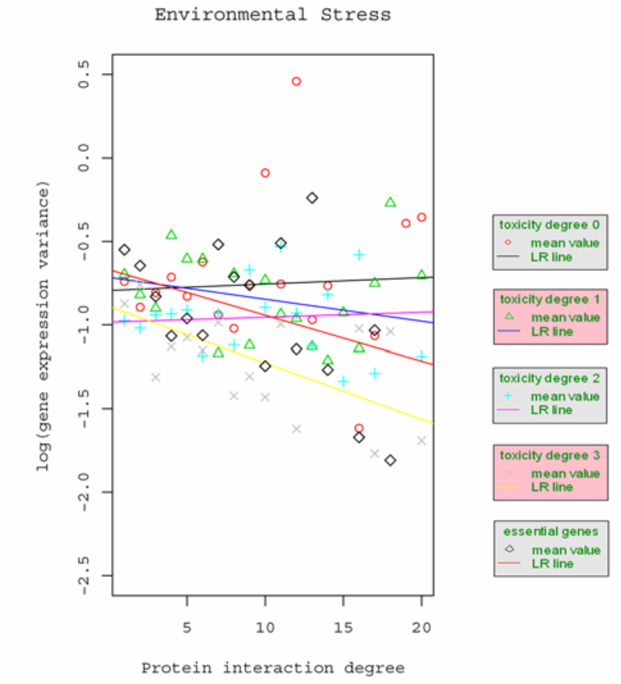

Supplemental Figure 15. The effect of toxicity degree, and protein interaction degree on gene expression variation. The mean expression variation and the linear regression fit to the expression variation of all the genes with respect to protein interaction degree for non-essential genes stratified according to toxicity degree and for the essential genes. The β values are 0.0039, -0.0131, 0.0030, -0.0337, and -0.0273 for toxicity degree 0, 1, 2, and 3, and the essential genes, respectively. The corresponding p-values are 0.6852, 0.1935, 0.7883, 0.0065, and 0.0507, respectively. R^2 is 0.03%, 0.44%, 0.03%, 5.25% and 2.03%, respectively. The labels are the same as those in Supplemental Figure 14.

Oxidative Stress

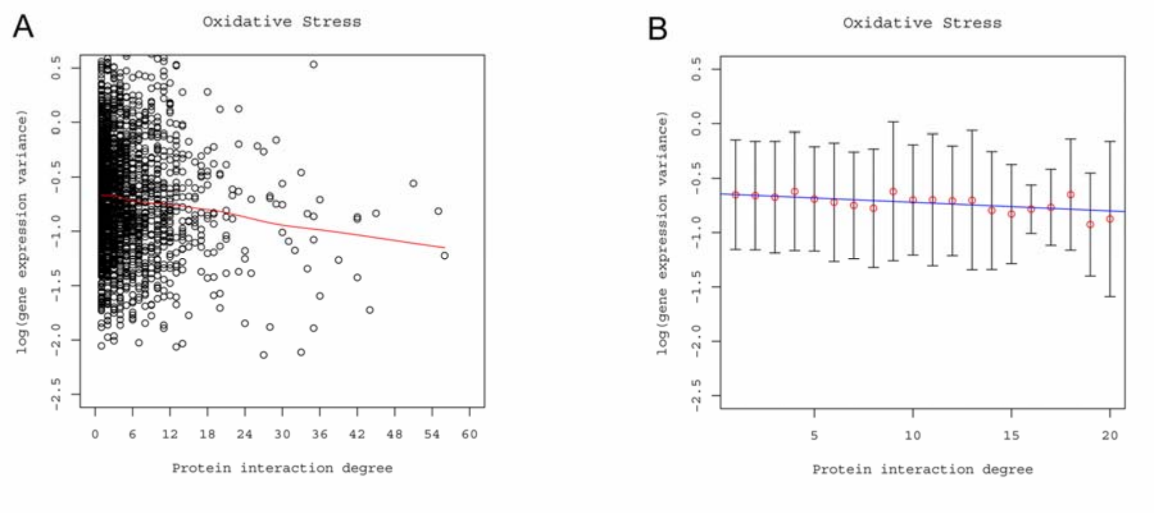

Supplemental Figure 16. Gene expression variation is negatively correlated with protein interaction degree. The x-axis represents protein physical interaction degree, and the y-axis represents the gene expression variation. A) The LOWESS fit to the gene expression variation. B) Bar-plot of the expression variation of all the genes with a given protein interaction degree together with the linear regression fit to the gene expression variation in relation to the interaction degree. The linear coefficient $\beta = -0.0080$, R^2 is 0.35% and the p-value = 0.0064. The red dots are the mean expression variation of the genes given the protein physical interaction (PPI) degree. The bar represents the standard deviation of the gene expression variation given PPI degree.

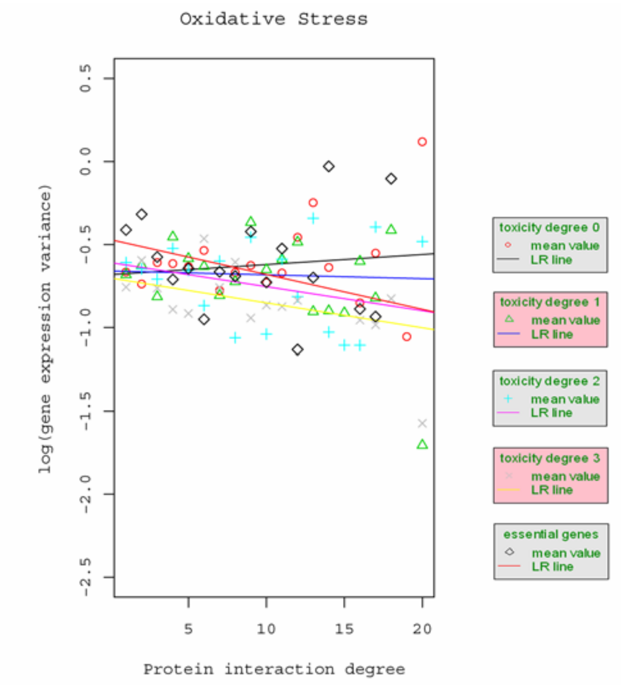

Supplemental Figure 17. The effect of toxicity degree, and protein interaction degree on gene expression variation. The mean expression variation and the linear regression fit to the expression variation of all the genes with respect to protein interaction degree for non-essential genes stratified according to toxicity degree and for the essential genes. The β values are 0.0061, -0.0023, -0.0145, -0.0150, and -0.0210 for toxicity degree 0, 1, 2, and 3, and the essential genes, respectively. The corresponding p-values are 0.3508, 0.7418, 0.1146, 0.1534, and 0.0446, respectively. R^2 is 0.15%, 0.03%, 1.05%, 1.47% and 2.14%, respectively. The labels are the same as those in Supplemental Figure 16.

Supplementary Figures Using the BioGrid Interaction Data Set

Ca and Na exposure

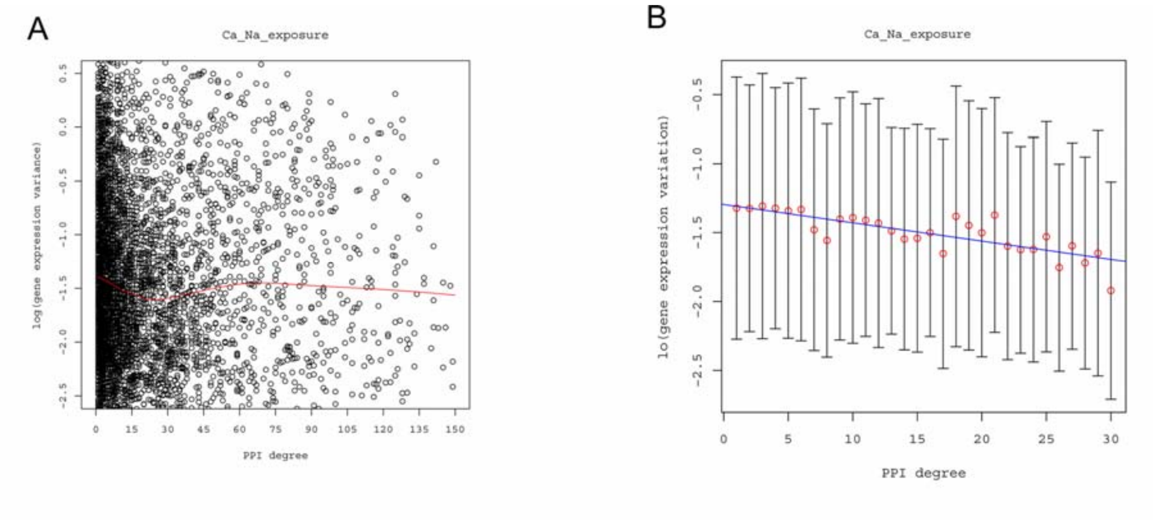

Supplemental Figure 18. Gene expression variation is negatively correlated with protein interaction degree. The x-axis represents protein physical interaction degree, and the y-axis represents gene expression variation. A) The LOWESS fit to the gene expression variation. B) Bar-plot of the expression variation of all the genes with a given protein interaction degree together with the linear regression fit to the gene expression variation in relation to the interaction degree. The linear coefficient β = -0.0133, R^2 = 1.36% and the p-value = 8.621e-13. The red dots are the mean expression variation of the genes given the protein physical interaction (PPI) degree. The bar represents the standard deviation of gene expression variation given PPI degree.

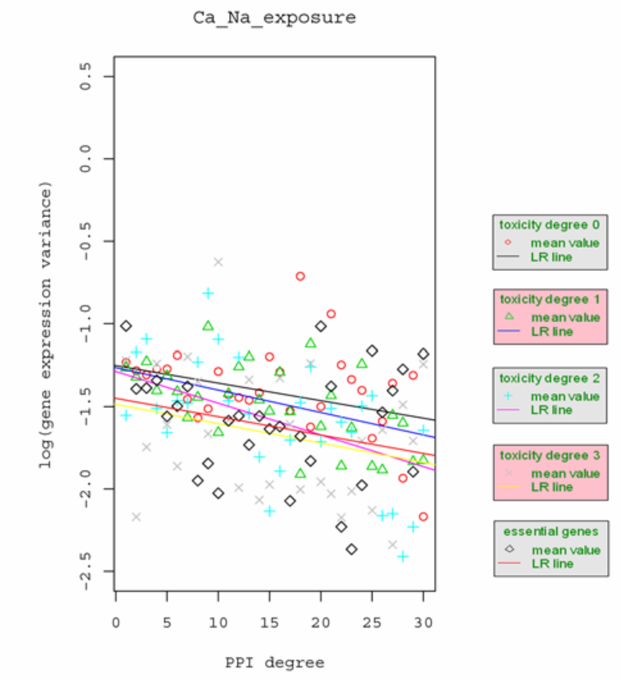

Supplemental Figure 19. The effect of toxicity degree, and protein interaction degree on gene expression variation. The mean expression variation and the linear regression fit to the expression variation of all the genes with respect to protein interaction degree for non-essential genes stratified according to toxicity degree and for the essential genes. The β values are -0.0106, -0.0136, -0.0192, -0.0118, and -0.0111 for toxicity degree 0, 1, 2, and 3, and the essential genes, respectively. The corresponding p-values are 0.0025, 0.0011, 0.0003, 0.2632, and 0.0925, respectively. R^2 is 0.69%, 1.26%, 4.04%, 1.23% and 1.42%, respectively. The labels are the same as those in Supplemental Figure 18.

Chemostat (nutritional stress)

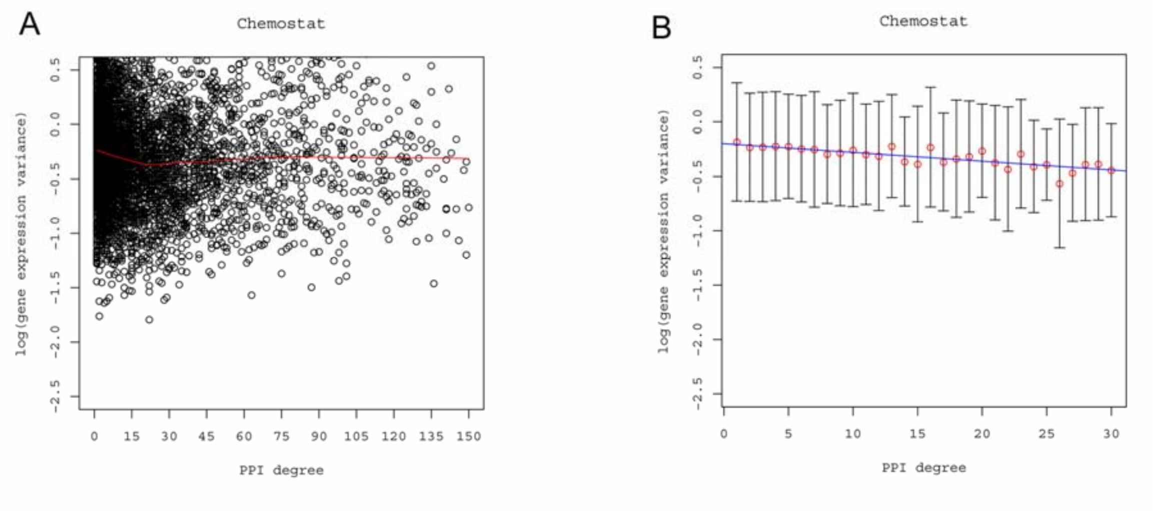

Supplemental Figure 20. Gene expression variation is negatively correlated with protein interaction degree. The x-axis represents protein physical interaction degree, and the y-axis represents gene expression variation. A) The LOWESS fit to the gene expression variation. B) Bar-plot of the expression variation of all the genes with a given protein interaction degree together with the linear regression fit to the gene expression variation in relation to the interaction degree. The linear coefficient $\beta = -0.0081$, $R^2 = 1.59\%$ and the p-value = 8.964e-15. The red dots are the mean expression variation of the genes given the protein physical interaction (PPI) degree. The bar represents the standard deviation of gene expression variation given PPI degree.

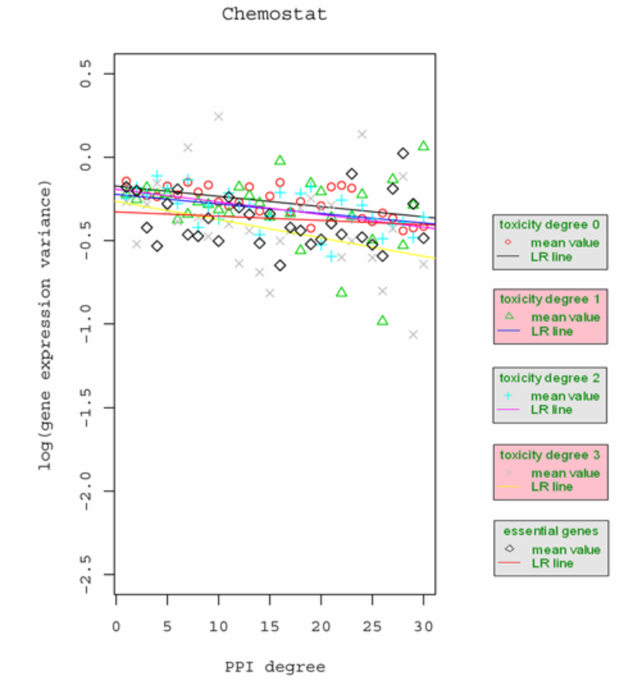

Supplemental Figure 21. The effect of toxicity degree, and protein interaction degree on gene expression variation. The mean expression variation and the linear regression fit to the expression variation of all the genes with respect to protein interaction degree for non-essential genes stratified according to toxicity degree and for the essential genes. The β values are -0.0062, -0.0057, -0.0076, -0.0110, and -0.0025 for toxicity degree 0, 1, 2, and 3, and the essential genes, respectively. The corresponding p-values are 0.0013, 0.0171, 0.0147, 0.0394, and 0.5151, respectively. R^2 is 0.77%, 0.68%, 1.86%, 4.1% and 0.21%, respectively. The labels are the same as those in Supplemental Figure 20.

Environmental Stress

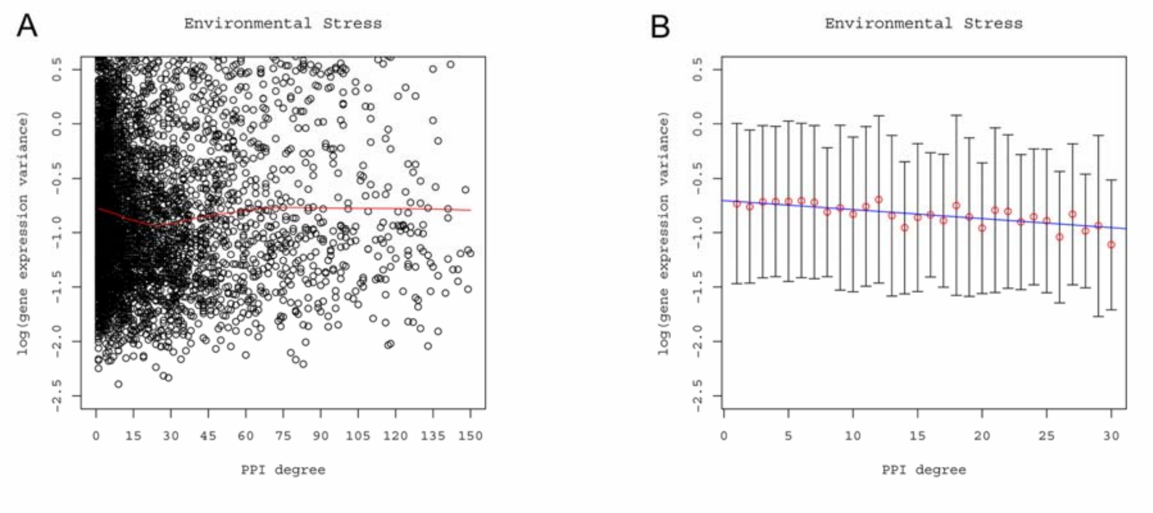

Supplemental Figure 22. Gene expression variation is negatively correlated with protein interaction degree. The x-axis represents protein physical interaction degree, and the y-axis represents gene expression variation. A) The LOWESS fit to the gene expression variation. B) Bar-plot of the expression variation of all the genes with a given protein interaction degree together with the linear regression fit to the gene expression variation in relation to the interaction degree. The linear coefficient $\beta = -0.0083$, $R^2 = 0.86\%$ and the p-value = 1.618e-08. The red dots are the mean expression variation of the genes given the protein physical interaction (PPI) degree. The bar represents the standard deviation of gene expression variation given PPI degree.

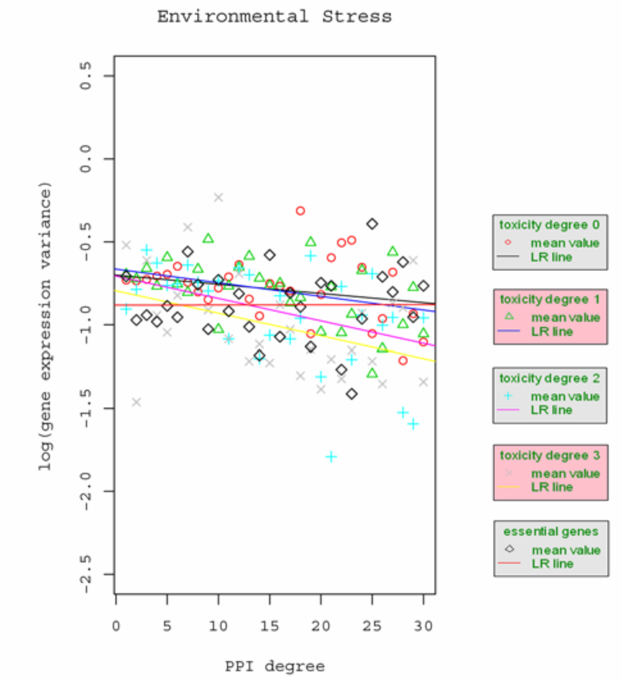

Supplemental Figure 23. The effect of toxicity degree, and protein interaction degree on gene expression variation. The mean expression variation and the linear regression fit to the expression variation of all the genes with respect to protein interaction degree for non-essential genes stratified according to toxicity degree and for the essential genes. The β values are -0.0055, -0.0082, -0.0135, -0.0135, and 0.0001 for toxicity degree 0, 1, 2, and 3, and the essential genes, respectively. The corresponding p-values are 0.0425, 0.0141, 0.0023, 0.0724, and 0.9834, respectively. R^2 is 0.31%, 0.72%, 2.92%, 3.13% and 0, respectively. The labels are the same as those in Supplemental Figure 22.

Oxidative Stress

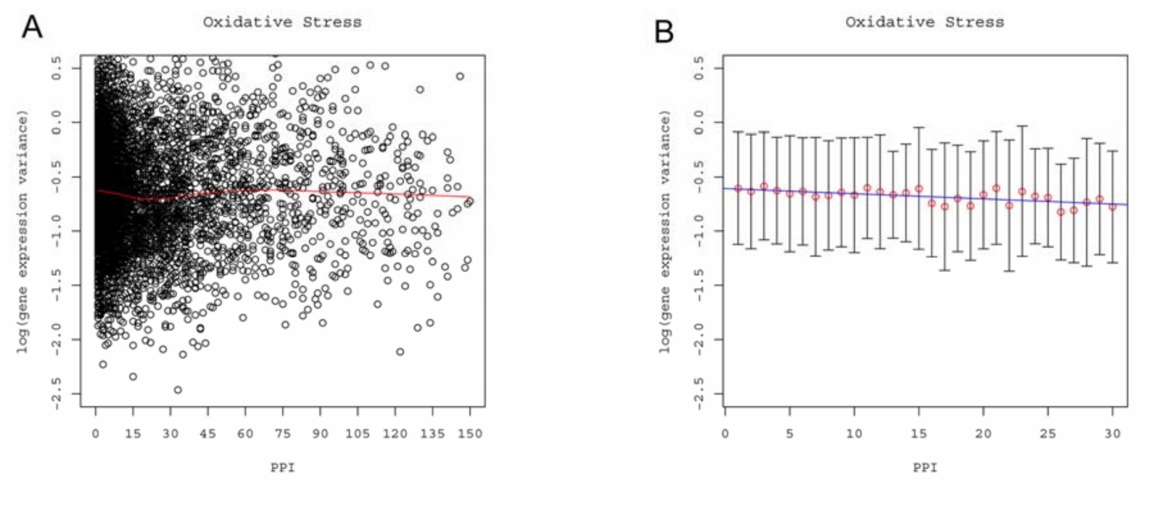

Supplemental Figure 24. Gene expression variation is negatively correlated with protein interaction degree. The x-axis represents protein physical interaction degree, and the y-axis represents the gene expression variation. A) The LOWESS fit to the gene expression variation. B) Bar-plot of the expression variation of all the genes with a given protein interaction degree together with the linear regression fit to the gene expression variation in relation to the interaction degree. The linear coefficient $\beta = -0.0048$, $R^2 = 0.53\%$ and the p-value = 8.107e-06. The red dots are the mean expression variation of the genes given the protein physical interaction (PPI) degree. The bar represents the standard deviation of the gene expression variation given PPI degree.

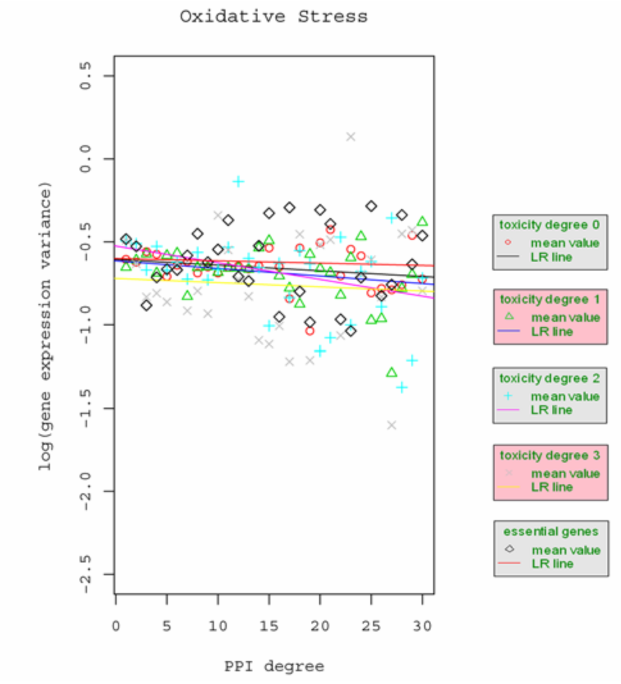

Supplemental Figure 25. The effect of toxicity degree, and protein interaction degree on gene expression variation. The mean expression variation and the linear regression fit to the expression variation of all the genes with respect to protein interaction degree for non-essential genes stratified according to toxicity degree and for the essential genes. The β values are -0.0034, -0.0046, -0.0101, -0.0025, and -0.0014 for toxicity degree 0, 1, 2, and 3, and the essential genes, respectively. The corresponding p-values are 0.0750, 0.0610, 0.0048, 0.6781, and 0.7446, respectively. R^2 is 0.24%, 0.42%, 2.49%, 0.17% and 0.05%, respectively. The labels are the same as those in Supplemental Figure 24.
