# Supplementary material for: The effects of protein interactions, gene essentiality and regulatory regions on expression variation
Source: BMC Syst Biol. 2008 Jun 26;2:54. doi: 10.1186/1752-0509-2-54 (PMC2474594; doi:10.1186/1752-0509-2-54)
Supplement: Additional file 2 — Overall analysis of four main factors (protein interaction degree, toxicity degree, number of TFs and the presence of TATA box) and their interactions. [file 1752-0509-2-54-S2.pdf]

Table of Contents

1. Model Selection using DIP protein interaction data with four expression data sets, respectively ……………………………….3

2. Model Selection using BioGrid protein interaction data with four expression data sets, respectively ……………………………….4

3. Model Selection using the average of expression variation across four expression data sets ……………………………………………...5

4. The effects of toxicity degree and # of TFs on expression variation (the average of expression variation across four expression data sets) stratified by the presence/absence of TATA box ………………6

5. The effects of toxicity degree on expression variation (the average of expression variation across four expression data sets) stratified by the set of environmental stress response (ESR)……………………..7

6. Model Selection using a combined expression data set ……..8

7. The effects of toxicity degree and # of TFs on expression variation using a combined expression data set stratified by the presence/absence of TATA box ……………………………………………………..9

8. The effects of toxicity degree on expression variation using a combined expression data set stratified by the set of environmental stress response (ESR) ……………….………………..….………10

9. The pairwise Spearman´s correlation between independent variables in the model ……………………………………………………11

Supplementary Table 1: Analysis of four factors and their interactions affecting expression variation using stepwise selection with AIC. The four factors include protein interaction degree (x1), toxicity degree (x2: treat essential genes as ones with toxicity degree 4), number of TFs (x3), and the presence of TATA box (x4: 1-TATA containing genes, 0-non-TATA containing genes). The protein interaction data used in this analysis is based on the DIP data set. The column marked with "√"indicates inclusion in the final linear model. The multiple linear regression is based on the final linear model, respectively. The p-value is related to the null hypothesis that $\beta \neq \mathbf{0}$ versus $\beta = \mathbf{0}$. R^2 is the variation explained by the model and each independent variable, respectively.

variable	Ca_Na_exposure			Chemostat			Environmental Stress			Oxidative Stress		
	model	p value	R^2	model	p value	R^2	model	p value	R^2	model	p value	R^2
x1				√	0.8892	0.003 %	√	0.0598	0.61%	√	0.1349	0.38%
x2	√	0.6756	0.03%	√	0.5487	0.06%	√	0.0059	0.13%			
x3	√	1.8e-09	6.02%	√	3.3e-9	5.86%	√	< 2e-16	14.49 %	√	0.0181	0.95%
x4	√	6.3e-07	4.17%	√	7.5e-05	2.67%	√	6.0e-12	7.82%	√	0.8492	0.006%
x1*x2												
x1*x3				√	0.0478	0.67%						
x1*x4										√	0.0364	0.75%
x2*x3												
x2*x4	√	0.0286	0.82%	√	0.0122	1.08%	√	0.0259	0.85%			
x3*x4	√	0.1199	0.41%	√	0.0736	0.55%	√	0.0002	2.37%			
R^2model	17.42%			12.83%			27.84%			3.45%		

Supplementary Table 2: Analysis of four factors and their interactions affecting expression variation using stepwise selection with AIC. The four factors include protein interaction degree (x1), toxicity degree (x2: treat essential genes as ones with toxicity degree 4), number of TFs (x3), and the presence of TATA box (x4: 1-TATA containing genes, 0-non-TATA containing genes). The protein interaction data used in this analysis is based on the BioGrid data set. The column marked with "√"indicates inclusion in the final linear model. The multiple linear regression is based on the final linear model, respectively. The p-value is related to the null hypothesis that $\beta \neq \mathbf{0}$ versus $\beta = \mathbf{0}$. R^2 is the variation explained by the model and each independent variable, respectively.

variable	Ca_Na_exposure			Chemostat			Environmental Stress			Oxidative Stress		
	model	p value	R^2	model	p value	R^2	model	p value	R^2	model	p value	R^2
x1	√	0.0050	0.72%	√	0.0002	1.29%	√	0.2220	0.14%	√	0.0154	0.53%
x2	√	0.1319	0.21%	√	0.3483	0.08%	√	0.8175	0.005 %			
x3	√	4.7e-16	5.87%	√	2.0e-5	1.65%	√	< 2e-16	12.72 %	√	9.6e-08	2.56%
x4	√	9.3e-14	4.96%	√	1.9e-9	3.25%	√	1.1e-12	4.55%	√	0.0003	1.19%
x1*x2												
x1*x3				√	0.0377	0.39%						
x1*x4							√	0.0281	0.44%			
x2*x3												
x2*x4	√	0.0152	0.54%	√	0.0131	0.56%	√	0.0229	0.47%			
x3*x4	√	0.0143	0.55%	√	0.0154	0.54%	√	2.2e-07	2.43%			
R^2model	18.62%			13.78%			26.23%			5.57%		

Supplementary Table 3: Analysis of four factors and their interactions affecting expression variation using stepwise selection with AIC. The four factors include protein interaction degree, toxicity degree, number of TFs, and the presence of TATA box. The protein interaction data used in this analysis is based on the MIPS, DIP and BioGrid data set. Gene expression variation is measured as the average of expression variation across four expression data sets including Ca_Na_exposure, Chemostat, Environmental Stress and Oxidative Stress. The column marked with "√"indicates inclusion in the final linear model. The multiple linear regression is based on the final linear model, respectively. The p-value is related to the null hypothesis that $\beta \neq \mathbf{0}$ versus $\beta = \mathbf{0}$. R^2 is the variation explained by the model and each independent variable, respectively.

variable	annotation	MIPS			DIP			BioGrid		
		model	p value	R^2	model	p value	R^2	model	p value	R^2
X1	Protein physical interaction degree	√	0.0166	0.46%	√	0.0852	0.51%	√	0.0004	1.13%
X2	Toxicity degree (treat essential genes as ones with toxicity degree 4)	√	0.8370	0.003%	√	0.0579	0.62%	√	0.2705	0.11%
X3	#TFs	√	< 2e-16	10.16%	√	1.3e-14	9.7%	√	1.8e-11	4.07%
X4	1-TATA containing genes, 0-non-TATA containing genes	√	< 2e-16	5.93%	√	3.4e-10	6.56%	√	< 2e-16	7.02%
x1*x2										
x1*x3								√	0.0910	0.26%
x1*x4										
x2*x3										
x2*x4		√	0.0026	0.73%	√	0.0025	1.56%	√	0.0027	0.82%
x3*x4		√	0.0005	0.97%	√	0.0360	0.75%	√	0.0045	0.74%
Total variation explained by the model		R^2=22.49%			R^2=24.679%			R^2=25.84%		

Supplementary Table 4. The effects of two factors on expression variation stratified by the presence/absence of TATA box. The linear model that includes toxicity degree and the number of TFs is built using the average of expression variation across four expression data sets including Ca_Na_exposure, Chemostat, Environmental Stress and Oxidative Stress. R^2 is the variation explained by the model and each independent variable, respectively. β is the linear coefficient in the linear model, the p-value is related to the null hypothesis that **β ≠ 0** versus **β = 0.**

TATA data set				
variables	annotation	β	p value	R^2
x1	Toxicity degree (treat essential genes as ones with toxicity degree 4)	-0.0744	0.0035	1.8%
x2	#TFs	0.0230	< 2e-16	13.79%
R^2	Total variation explained by the model	15.03%		
**Non-TATA data set**				
variables	annotation	β	p value	R^2
x1	Toxicity degree (treat essential genes as ones with toxicity degree 4)	0.0018	0.8588	0.003%
x2	#TFs	0.0362	< 2e-16	16.25%
R^2	Total variation explained by the model	16.28%		

Supplementary Table 5. The effects of toxicity degree on expression variation stratified by the set of environmental stress response (ESR). The linear model is built using the average of expression variation across four expression data sets including Ca_Na_exposure, Chemostat, Environmental Stress and Oxidative Stress. R^2 is the variation explained by the model. β is the linear coefficient in the linear model, the p-value is related to the null hypothesis that **$\beta \neq 0$** versus **$\beta = 0$.**

Gene group	β	p-value
ESR	-0.0327	0.0330
Non-ESR	-0.0425	2.20e-13

Supplementary Table 6. Analysis of four factors and their interactions affecting expression variation using stepwise selection with AIC. The four factors include protein interaction degree, toxicity degree, number of TFs, and the presence of TATA box. The protein interaction data used in this analysis is based on the MIPS, DIP and BioGrid data set. Gene expression variation is variability of gene expression across more than 1,500 conditions using a combined data set. The column marked with "√"indicates inclusion in the final linear model. The multiple linear regression is based on the final linear model, respectively. The p-value is related to the null hypothesis that $\beta \neq \mathbf{0}$ versus $\beta = \mathbf{0}.$ R^2 is the variation explained by the model and each independent variable, respectively.

variable	annotation	MIPS			DIP			BioGrid		
		model	p-value	R^2	model	p-value	R^2	model	p-value	R^2
X1	Protein physical interaction degree	√	0.0027	0.76%	√	0.453	0.73%	√	0.1309	0.22%
X2	Toxicity degree (treat essential genes as ones with toxicity degree 4)	√	0.1692	0.16%	√	0.9462	0.001 %	√	0.0126	0.061%
X3	#TFs	√	< 2e-16	14.52%	√	8.2e-13	16.61 %	√	< 2e-16	12.74%
X4	1-TATA containing genes, 0-non-TATA containing genes	√	< 2e-16	12.72%	√	< 2e-16	8.92%	√	< 2e-16	12.76%
x1*x2										
x1*x3										
x1*x4					√	0.0916	0.52%			
x2*x3										
x2*x4		√	0.0012	0.88%	√	0.0019	1.75%	√	0.0045	0.78%
x3*x4		√	0.0015	0.85%	√	0.0029	1.61%	√	0.0038	0.82%
Total variation explained by the model		R^2 = 37.53%			R^2 = 40.99%			R^2 = 38.65%		

Supplementary Table 7. The effects of two factors on expression variation stratified by the presence/absence of TATA box. The linear model that includes toxicity degree and the number of TFs is built using gene expression variability across more than 1,500 conditions on a combined data set. R^2 is the variation explained by the model and each independent variable, respectively. β is the linear coefficient in the linear model, the p-value is related to the null hypothesis that $\beta \neq \mathbf{0}$ versus $\beta = \mathbf{0}$

TATA data set				
variables	annotation	β	p value	R^2
x1	Toxicity degree (treat essential genes as ones with toxicity degree 4)	-0.1060	9.63e-05	3.55%
x2	#TFs	0.0274	< 2e-16	18.76%
R^2	Total variation explained by the model	21%		
**Non-TATA data set**				
variables	annotation	β	p value	R^2
X1	Toxicity degree (treat essential genes as ones with toxicity degree 4)	-0.0193	0.1012	0.24%
x2	#TFs	0.0398	< 2e-16	15.17%
R^2	Total variation explained by the model	15.56%		

Supplementary Table 8. The effects of toxicity degree on expression variation stratified by the set of environmental stress response (ESR). The linear model is built using gene expression variability across more than 1,500 conditions on a combined data set. R^2 is the variation explained by the model. β is the linear coefficient in the linear model, the p-value is related to the null hypothesis that $\beta \neq \mathbf{0}$ versus $\beta = \mathbf{0}$

Gene group	β	p-value
ESR	-0.0428	0.0085
Non-ESR	-0.0687	<2e-16

Supplementary Table 9. The pairwise Spearman´s correlation between independent variables in each model with different protein interaction data. The ρ value is Spearman´s correlation coefficient and p-value is related to the null hypothesis that ρ ≠ **0** versus ρ = **0.**

	Toxicity degree		# TFs		TATA	
	ρ	p value	ρ	p value	ρ	p value
Protein interaction degree (MIPS)	0.1558	<0.0001	-0.0773	0.0042	-0.0823	0.0023
Toxicity degree			-0.0697	0.0098	-0.1574	<0.0001
# TFs					0.2221	<0.0001

	Toxicity degree		# TFs		TATA	
	ρ	p value	ρ	p value	ρ	p value
Protein interaction degree (DIP)	0.1530	0.0001	-0.0797	0.0482	-0.0973	0.0158
Toxicity degree			-0.1557	0.0001	-0.1913	<0.0001
# TFs					0.2879	<0.0001

	Toxicity degree		# TFs		TATA	
	ρ	p value	ρ	p value	ρ	p value
Protein interaction degree (BioGrid)	0.2455	<0.0001	0.0529	0.0427	-0.1541	0.0001
Toxicity degree			-0.0466	0.0747	-0.1650	<0.0001
# TFs					0.2237	<0.0001
